# Automated Characterization and Parameter-Free Classification of Cell Tracks Based on Local Migration Behavior

**DOI:** 10.1371/journal.pone.0080808

**Published:** 2013-12-06

**Authors:** Zeinab Mokhtari, Franziska Mech, Carolin Zitzmann, Mike Hasenberg, Matthias Gunzer, Marc Thilo Figge

**Affiliations:** 1 Applied Systems Biology, HKI-Center for Systems Biology of Infection, Leibniz-Institute for Natural Product Research and Infection Biology – Hans-Knöll-Institute (HKI), Jena, Germany; 2 Friedrich Schiller University Jena, Germany; 3 German Rheumatism Research Centre (DRFZ), Berlin, Germany; 4 Centre for Medical Biotechnology, University Duisburg-Essen, Germany; Universitat Pompeu Fabra, Spain

## Abstract

Cell migration is the driving force behind the dynamics of many diverse biological processes. Even though microscopy experiments are routinely performed today by which populations of cells are visualized in space and time, valuable information contained in image data is often disregarded because statistical analyses are performed at the level of cell populations rather than at the single-cell level. Image-based systems biology is a modern approach that aims at quantitatively analyzing and modeling biological processes by developing novel strategies and tools for the interpretation of image data. In this study, we take first steps towards a fully automated characterization and parameter-free classification of cell track data that can be generally applied to tracked objects as obtained from image data. The requirements to achieve this aim include: (i) combination of different measures for single cell tracks, such as the confinement ratio and the asphericity of the track volume, and (ii) computation of these measures in a staggered fashion to retrieve local information from all possible combinations of track segments. We demonstrate for a population of synthetic cell tracks as well as for *in vitro* neutrophil tracks obtained from microscopy experiment that the information contained in the track data is fully exploited in this way and does not require any prior knowledge, which keeps the analysis unbiased and general. The identification of cells that show the same type of migration behavior within the population of all cells is achieved via agglomerative hierarchical clustering of cell tracks in the parameter space of the staggered measures. The recognition of characteristic patterns is highly desired to advance our knowledge about the dynamics of biological processes.

## Introduction

Image-based systems biology is a growing field of research that involves the development of methods for the quantitative analysis and modeling of information contained in microscopic images. Today, investigations of biological processes are often routinely accompanied by microscopy experiments, however, in many cases the acquired image data are eventually only used for illustrative purposes. The disregard of valuable information that is contained in these data is partly a consequence of lacking strategies for their appropriate analysis [1].

In order to capture important details of a biological process under consideration and to arrive at quantitative predictions, it is generally required that algorithms capable of analyzing the specific experimental data have to be developed first [2], [3]. In particular, algorithms for the automated characterization and parameter-free classification of cell tracks at the single-cell level are currently lacking. For example, while the time-dependent positions of cells are recorded in microscopy experiments at the single-cell level, in many cases the subsequent analysis is performed by statistical means at the level of the cell population, where the absolute cell positions in the biological sample and the relative temporal offset between cell tracks are integrated out. In fact, analyzing image data obtained at the single-cell level by statistical means at the level of the cell population may strongly reduce the predictive power of the analysis and may possibly even lead to incorrect conclusions with regard to spatio-temporal changes in the cellular migration behavior.

A prominent example concerns the interpretation of early experimental studies on B cell migration in germinal centers, where the cellular migration behavior was evaluated by statistical analyses of cell populations from which it was predicted to be purely random [4]–[6]. However, applying an image-based systems biology approach, it was first observed by Figge *et al.* [7] and subsequently confirmed by Beltman *et al.* [8] that the experimental data are compatible with germinal center B cell tracks containing combinations of random and directed track segments. Computer simulations based on the image data suggested that these directed segments could be induced by transient chemotaxis and it was postulated that directed segments in the cell tracks are a necessary condition to reconcile the observed B cell migration behavior with the peculiar zonal morphology in germinal centers [7], [9]. In other words, the cellular migration behavior was predicted to be dependent on the cell position in the biological sample with cells changing from a more random to a more directed mode of migration at the germinal center zone boundary. Naturally, the tailored cell track analysis by Beltman *et al.* [8] exploited this pre-existing hypothesis on transient chemotaxis at the zone boundary as well as additional prior knowledge with respect to the estimated position of the germinal center zone boundary from experiment. In general, however, it would be preferable to perform the characterization and classification of the cellular migration behavior in a fully automated fashion avoiding the use of any prior knowledge.

The importance of analysing the migration behavior of single cells was realized in recent microscopy experiments of cell migration, *e.g.* for the guidance of dendritic cells by haptotactic chemokine gradients towards lymphatic vessels [10], for B cell trafficking within the T cell area to enter follicles in the lymph node [11], and for neutrophil migration directed by inflammatory chemokines [12]. The chemokine dependent migration of neutrophils was shown to be influenced by various factors such as the distance to the site of infection. Furthermore, the migration was described to be of random walk type with directed behavior when affected by chemokine gradients. Thus, neutrophil migration shows a rich diversity of different types of migration behavior emphasizing once again the need for single cell track analyses.

In this work, we take first steps towards a fully automated characterization and parameter-free classification of cell track data that can be generally applied to tracked objects as obtained from image data. We identified the following two requirements to achieve this aim: (i) combination of different measures such as the confinement ratio and the asphericity of the cell track volume and (ii) computation of these measures in a staggered fashion to retrieve local information from all possible track segments. In this way, the information contained in the cell track data is exploited while drawing on prior knowledge is deliberately abandoned in order to keep the analysis unbiased and general. The recognition of characteristic patterns is of high interest. We demonstrate that this can be achieved via hierarchical clustering of cell tracks in the parameter space related to the confinement ratio and the volume asphericity.

## Methods

This section summarizes the mathematical characterization of cell tracks at the level of single cells and the classification of cell tracks into different types of migration behavior.

### Cell track characterization

The characterization of cell tracks at the level of single cells is usually achieved by calculating characteristic measures, *e.g.* the confinement ratio, which are computed along the track of a cell relative to its initial position. Referring to these quantities as linear measures when computed as a function of time, they are extended for cell track characterization in the following way: (i) characteristic measures are computed as staggered quantities, *i.e.* as a function of time and for a varying starting time points along the track, (ii) staggered measures are studied in a combined fashion, and (iii) these measures are computed both in the presence and absence of time-ordering with regard to cell positions. It should be noted that, in contrast to earlier studies [13], we keep the time resolution unchanged in the computation of the staggered measures.

In general, a population of 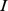 cells in a 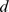-dimensional spatial environment at time point 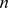 is defined by the cell position vectors,
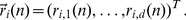
(1)


Here, 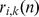 denotes the 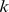
th dimension (with 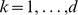) of the position vector for the cell with identification number 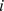 (with 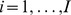) and 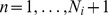 refers to the number of time points 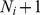 of this cell. Assuming that cell positions are measured at constant time intervals 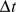, the track of the 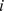
th cell is uniquely defined by the time-ordered sequence 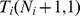 of its position vectors:
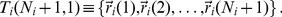
(2)


Note that 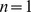 refers to the time point at which the cell is observed in the system for the first time, *i.e.* 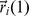 denotes the initial position of the 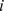
th cell.

**Figure 1:**
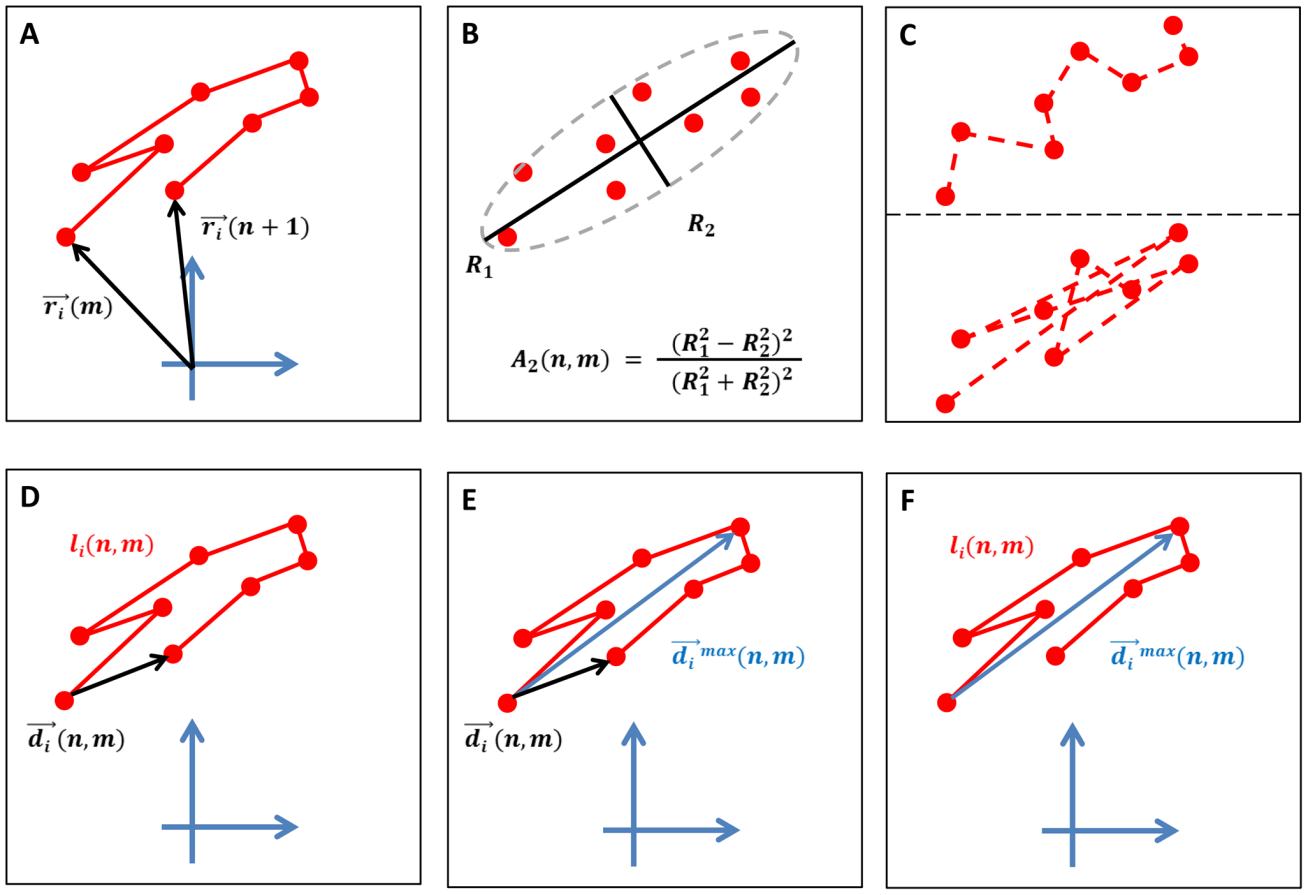
Schematic cell track characterization. *(A)* Example of a cell track segment. *(B)* The volume asphericity is determined by the cell positions of the track segment that are viewed as uncorrelated data points. *(C)* Track segments with different time-orderings are compatible with one and the same volume, including time-ordering based on closest data points (top) and time-ordering based on farthest data points (bottom). *(D)* The confinement ratio is determined by the displacement over the length of the cell track segment. *(E)* The displacement ratio is determined by the displacement over the maximal displacement of the cell track segment. *(F)* The outreach ratio is determined by the maximal displacement over the length of the cell track segment.

The calculation of measures as staggered quantities implies that computations are not based on Eq. (2) alone, but rather that all possible combinations
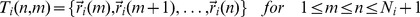
(3)


of a cell track's time-ordered segments are considered. An example for a cell track segment is depicted in Fig. 1A. While standard analyses of cell tracks by linear measures, *e.g.* based on the confinement ratio, are performed for 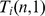 and the corresponding number of function values scales with 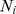, computations in a staggered fashion imply that the number of function values scales with 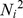 for combinatorial reasons. Though computationally more expensive, the advantage of this procedure is that it allows identifying the transient characteristics of cell tracks. Taking these transient characteristics into account turns out to be a necessary requirement for the correct classification of cell tracks into different types of migration behavior.

Leaving the time-ordering of cell positions along the track segment out of consideration, cell positions of a track segment are not viewed as subsequent data points but rather as a cloud of uncorrelated data points. From this point of view the track segment 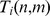 is represented by the gyration tensor 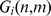, which defines the 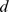-dimensional ellipsoidal volume that resembles the shape of this cloud of data points [14], [15]. The gyration tensor is a symmetric 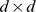 matrix with entries,
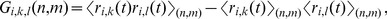
(4)


where 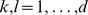 are labels for the cartesian coordinates of the 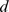-dimensional spatial system. The averages are taken over consecutive cell positions 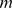 to 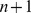 (with 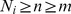) for the track segment of the 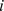
th cell:

The averaging procedure is defined such that at least two cell positions are involved, *i.e.* diagonal elements 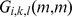 refer to cell positions 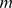 and 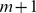. The 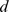 eigenvalues of the gyration tensor 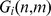 correspond to the squares of the gyration radii 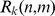 (with 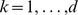) defining the 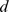-dimensional ellipsoidal volume of track segment 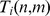. It should be noted, however, that the uniquely defined 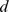-dimensional ellipsoidal volume 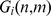 of track segment 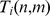 is compatible with 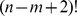 different combinations of time-orderings in the cellular positions.

#### Staggered volume asphericity

The shape of the track volume is characterized by universal quantities such as the asphericity, 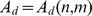, which quantifies the deviation of the track volume from a 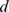-dimensional sphere (with 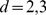). It is expressed in terms of the gyration radii 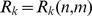, *i.e.* the square-roots of the 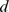 eigenvalues of the gyration tensor Eq. (4), and is given by [15]:
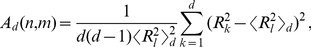
(6)where the average value
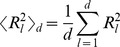
(7)


**Table:** Table 2. Properties of neutrophil cell tracks per migration type for 2D clustering.

average properties	type 1:	type 2:	type 3:
	fairly straight	strongly confined	purely random
staggered confinement ratio	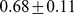	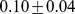	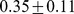
staggered volume asphericity	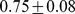	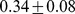	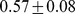
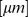 length of cell tracks	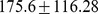	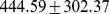	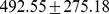
number of time points	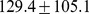	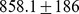	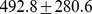
number of cell tracks	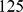	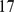	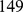
staggered confinement ratio	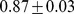	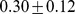	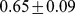
staggered outreach ratio	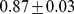	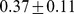	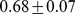
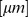 length of cell tracks	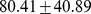	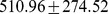	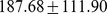
number of time points	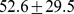	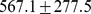	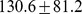
number of cell tracks	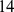	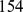	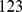
staggered confinement ratio	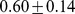	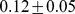	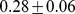
staggered displacement ratio	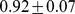	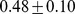	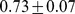
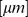 length of cell tracks	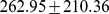	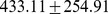	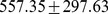
number of time points	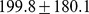	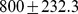	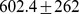
number of cell tracks	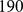	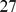	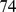
staggered volume asphericity	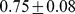	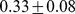	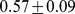
staggered outreach ratio	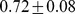	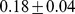	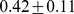
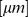 length of cell tracks	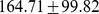	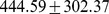	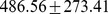
number of time points	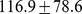	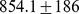	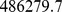
number of cell tracks	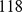	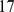	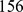
staggered volume asphericity	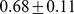	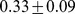	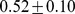
staggered displacement ratio	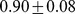	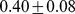	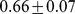
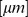 length of cell tracks	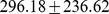	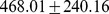	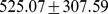
number of time points	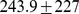	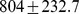	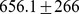
number of cell tracks	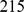	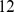	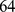
staggered outreach ratio	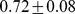	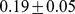	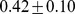
staggered displacement ratio	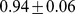	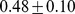	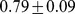
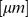 length of cell tracks	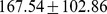	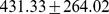	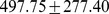
number of time points	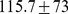	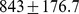	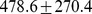
number of cell tracks	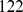	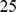	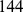

For each average staggered measure the values are highest (lowest) in the case of type 1 (type 2) cell tracks, while cell tracks of type 3 always assume intermediate values. These results are shown together with the cell track length, number of time points and number of neutrophils per migration type for all possible combinations of 2D clustering. The Wilcoxon rank-sum test revealed that the average staggered measures of different sub-populations were significantly different (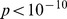).

is taken in the 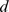-dimensional space. It can be easily shown that the value of the asphericity is restricted to 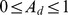. In case all gyration radii are identical, 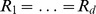, the track volume corresponds to a 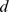-dimensional sphere, which is reflected by vanishing asphericity: 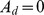. This distribution of data points may be interpreted as originating from the track of a cell that performs random migration covering the space isotropically. In contrast, a perfectly straight cell track implies that all but one 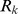 equal zero, such that 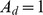 is indicative for the maximal deviation of the track volume from the 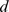-dimensional sphere.

**Table 3:** Properties of neutrophil cell tracks per migration type for 3D clustering.

average properties	type 1:	type 2:	type 3:
	fairly straight	strongly confined	purely random
staggered confinement ratio	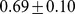	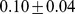	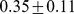
staggered volume asphericity	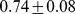	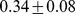	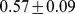
staggered outreach ratio	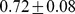	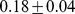	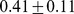
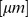 length of cell tracks	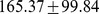	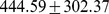	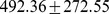
number of time points	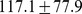	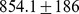	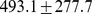
number of cell tracks	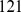	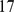	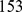
staggered confinement ratio	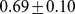	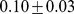	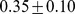
staggered volume asphericity	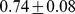	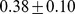	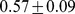
staggered displacement ratio	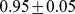	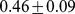	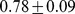
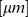 length of cell tracks	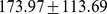	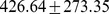	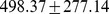
number of time points	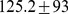	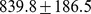	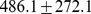
number of cell tracks	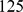	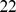	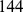
staggered confinement ratio	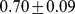	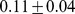	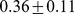
staggered outreach ratio	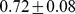	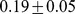	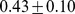
staggered displacement ratio	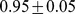	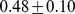	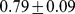
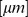 length of cell tracks	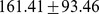	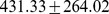	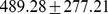
number of time points	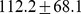	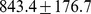	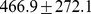
number of cell tracks	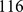	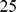	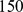
staggered volume asphericity	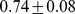	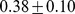	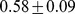
staggered outreach ratio	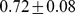	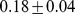	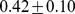
staggered displacement ratio	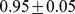	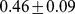	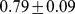
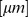 length of cell tracks	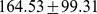	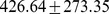	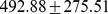
number of time points	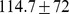	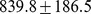	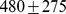
number of cell tracks	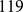	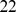	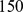

For each average staggered measure the values are highest (lowest) in the case of type 1 (type 2) cell tracks, while cell tracks of type 3 always assume intermediate values. These results are shown together with the cell track length, number of time points and number of neutrophils per migration type for all possible combinations of 3D clustering. The Wilcoxon rank-sum test revealed that the average staggered measures of different sub-populations were significantly different (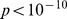).

The volume asphericity is illustrated in Fig. 1B, where the lack of time-ordering of cell positions along the track segment gives rise to 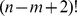 different combinations that are compatible with the same ellipsoidal volume, of which two extreme examples are shown in Fig. 1C.

**Table 4:** Properties of neutrophil cell tracks per migration type for 4D clustering.

average properties	type 1:	type 2:	type 3:
	fairly straight	strongly confined	purely random
staggered confinement ratio	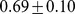	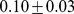	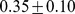
staggered volume asphericity	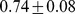	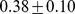	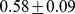
staggered outreach ratio	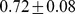	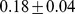	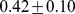
staggered displacement ratio	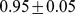	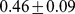	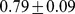
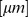 length of cell tracks	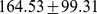	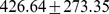	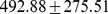
number of time points	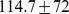	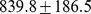	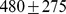
number of cell tracks	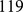	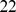	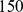

For each average staggered measure the values are highest (lowest) in the case of type 1 (type 2) cell tracks, while cell tracks of type 3 always assume intermediate values. These results are shown together with the cell track length, number of time points and number of neutrophils per migration type for 4D clustering. The Wilcoxon rank-sum test revealed that the average staggered measures of different sub-populations were significantly different (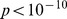).

#### Staggered volume prolateness

In three spatial dimensions (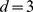), the shape of the track volume is further characterized by a universal quantity referred to as prolateness. Intermediate values of the asphericity, 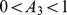, refer to ellipsoidal volumes that may be prolate with 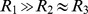 – *i.e.* corresponding to a cigar-shaped volume along one spatial direction with confinement in the other two spatial dimensions – or oblate with 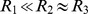 – *i.e.* corresponding to a random cell track in two spatial dimensions with confinement along the third spatial direction. The prolateness 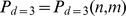 of the three-dimensional track volume is given by [15]:
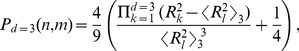
(8)with 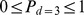. Inspection of this formula reveals that 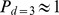 for prolate track volumes, while for oblate track volumes 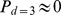. Spherical track volumes yield intermediate values around 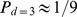. In passing we note that – for obvious reasons – the prolateness does not represent a meaningful measure in dimensions 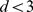.

#### Staggered confinement ratio

A measure for the confinement of a cell track that does respect the time-ordering in the sequence of cell positions is given by the confinement ratio [16]. This quantity compares the time-dependent length of the cell track with that of the corresponding displacement vector, as is schematically shown in Fig. 1D.

The displacement vector between time points 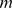 and 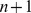 in terms of the cell position vectors Eq. (1) is given by
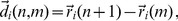
(9)where we assume without loss of generality that 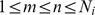. The track length between time points 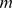 and 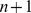 can be represented as

in terms of the displacement vector 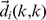 that refers to subsequent time points 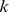 and 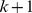. The staggered confinement ratio is then defined as the ratio of these two quantities,

Viewing 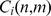 as entries of the 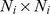 matrix 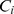, we note that this matrix is symmetric because both the displacement vector 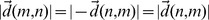 and the track-segment length 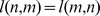 are invariant under the time reversal operation 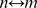 such that 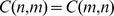. Furthermore, the diagonal elements of 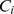 take values 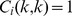 because 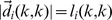 for all 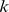. In general, 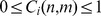, since
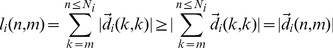
(12)according to the triangle inequality 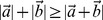 for real vectors 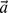 and 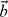.

#### Staggered displacement ratio

The staggered displacement ratio is depicted in Fig. 1E and is a variation of the staggered confinement ratio. Here, the length of the cell track is replaced by the length of the longest displacement vector, 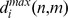, among all possible pair combinations 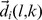 for the displacement vector between time points 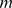 and 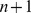 (with 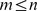):
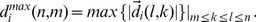
(13)


The staggered displacement ratio is then given by
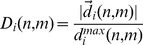
(14)and corresponds to an extension of the previously applied quantity 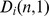 for the whole cell track [17]. For a perfectly straight track, we obtain 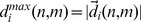 and 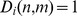, while in general the staggered displacement ratio takes values 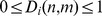. In passing we note that the displacement ratio shares the symmetry property 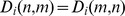 with the other staggered measures.

Note that the staggered displacement ratio 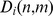 and the staggered confinement ratio 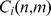 represent different aspects of a cell track. Assuming that cells are not running on the spot, the length of a cell track always increases with the next time step. In contrast, except for perfectly straight tracks where 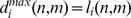, the displacement vector with maximal length does in general not increase continuously but may remain constant over several time steps. Thus, while the confinement ratio measures the displacement length relative to the track length, the reference in the displacement ratio is set by the two most distant cell positions in the track segment under consideration.

#### Staggered outreach ratio

We define the staggered outreach ratio,
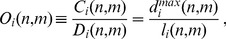
(15)by combining the staggered confinement ratio 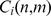 and the staggered displacement ratio 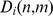. This symmetric measure, 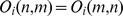, has the intuitive meaning that the length of a track segment allows for a maximal displacement within the track segment. Thus, as shown in Fig. 1F, while the confinement ratio compares the direct path between track points at time points 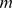 and 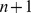 with the corresponding length of the track segment, the outreach ratio refers to the maximal displacement length that is realized within this track segment.

### Cell track classification

The classification of cell tracks according to their type of migration behavior was realized by adopting the method of hierarchical clustering from the field of data mining, which is routinely applied to discover relevant patterns within large data sets [18]. In the present context, hierarchical clustering of a population of cell tracks is performed to identify sub-populations of cell tracks that show similar migration behavior within a continuum of observed behaviors.

The advantages of hierarchical clustering over other clustering methods are that it is a generic approach with regard to the set of unlabelled data points and that it is parameter-free. Hierarchical clustering methods do not require any prior knowledge, *e.g.* on the expected number of relevant clusters, since they are solely based on a given distance measure. The comparison of distances between groups of data points determines the strongest relation between any two groups according to a distance criterion and a hierarchy of clusters emerges by sequentially relating groups of data points. Visualization of the corresponding nested structure in terms of a distance-based dendrogram finally allows identifying clusters of data points with related properties.

In general, all possible cluster configurations could be inferred from the corresponding dendogram and in this way the whole continuum of migration behavior in the cell track data could be analyzed. In practice, we focused on the analysis of extreme types of migration behavior, *e.g.* fairly straight cell tracks and strongly confined cell tracks. To this end we mainly considered three clusters allowing for the existence of mixed cell tracks in between these two extremes. Whether or not the three identified clusters yield a meaningful classification of the cell migration behavior depends on the necessary condition that these clusters have significantly different characteristic measures.

We applied the method of agglomerative hierarchical clustering, *i.e.* the initial number of clusters equals the number of data points and these clusters are sequentially grouped into larger clusters following a bottom-up strategy. We represented cell tracks as data points in the parameter space that is defined by the average values of the staggered measures using the Euclidean distance metric. Groups of data points were represented by their centroid position that was obtained by averaging over the position of associated data points and the number of relevant clusters was inferred from the corresponding dendrogram. This enabled us to identify clusters of cell tracks with comparable type of migration behavior. To test for the necessary condition that identified clusters have significantly different characteristic measures, we first checked whether these values obtained from a cluster's cell tracks were normally distributed. This was achieved by performing the Shapiro-Wilk test and for sufficiently large 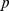-values (*e.g.*, 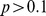) the data were considered to be normally distributed. In this case, the average values of a characteristic measure for two clusters were tested for significant difference using Welch's t-test. However, in most cases the Shapiro-Wilk test revealed that the data were not normally distributed and a Wilcoxon rank-sum test was performed to test for a significant difference in the average values of the characteristic measure.

#### Cell track data

To illustrate the information contained in the staggered measures of cell tracks, we generated and analyzed synthetic cell track data of specific types in three spatial dimensions. The analysis was extended to *in vitro* experiments on neutrophil migration in two spatial dimensions to demonstrate that real cell track data obtained from manually tracked time-lapse data generated by confocal laser scanning microscopy can be classified into different types of migration.

#### Migration of synthetic cells

Synthetic cell tracks were generated by a self-written computer algorithm focusing on three different types: fairly straight cell tracks (type 1), strongly confined cell tracks (type 2), and purely random cell tracks (type 3). It should be noted that the description of migration types has to be taken with some care, because this will depend on the spatial and temporal scale of the microscopy experiment, respectively, on the size of the field of view and on the imaging time.

Each cell track was generated in a three-dimensional spatial environment for 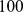 time points with time step 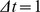 min. In each time step, a new speed value 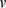 and turning angle 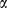 were randomly chosen. It should be noted that 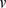 and 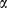 define, respectively, the slant height and the opening angle of a cone and the new cell position was located at a randomly chosen position on the rim of this cone.

The instantaneous cell speed 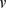 was drawn from the normal distribution
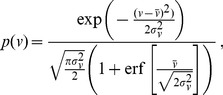
(16)where 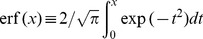 denotes the error function that ensures normalization of Eq. (16) over the whole speed range 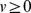. We set the average speed 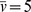 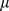
m/min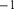 and the standard deviation 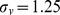 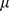
m/min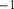.

The turning angle 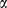 was drawn on the interval 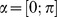 from the normal distribution
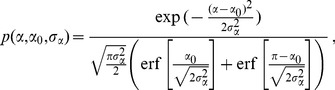
(17)where the center 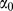 and the width 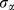 of the Gaussian were adjusted for the different types of cell tracks. In the case of type 1 cell tracks, we set 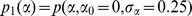, implying that cell tracks became fairly straight since average turning angles were of the relatively small size 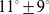 (see Fig. S1). In contrast, cell tracks of type 2 were generated from the distribution 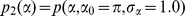, with the typical size of average turning angles around 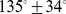, *i.e.* turns per time step were relatively large and cell tracks became strongly confined in space (see Fig. S2). For cell tracks of type 3, we set 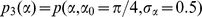, resulting into average turning angles of intermediate size 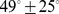 (see Fig. S3).

A population of synthetic cell tracks was composed of three sub-populations with fractions 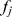, where 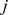 refers to the type of migration behavior and 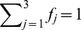. Choosing the population size to be 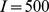 cell tracks with 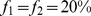 and 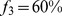, the combined turning angle distribution 
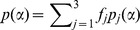
 is characterized by the average turning angle 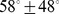.

#### Migration of neutrophils

*In vitro* experiments on neutrophil migration were performed to generate microscopic image data that were analyzed by manual cell tracking. All cells used in this study were isolated from organs of mice. Before organ harvest the mice were sacrificed painlessly by deep narcosis with isoflurane anesthetics followed by cervical dislocation according to institutional guidelines and no invasive procedures were carried out using live animals. According to the German Tierschutzgesetz (TSchG) the use of animal tissue following painless sacrifice and without any further treatment of live animals is not considered an animal experiment and therefore does not require ethical approval. However, the animal welfare officer was informed and had to collect information on the number of animals used for tissue donation. This information was forwarded to the local authorities (LANUV, Nordrhein Westfalen). The neutrophils were isolated from the four hind leg long bones of a C57/BL6 mouse as previously described in detail [19]. Briefly, bone marrows were flushed from the bones with a syringe, and transformed into single cell suspensions by pipetting. Single cell suspensions were subjected first to two rounds of osmotic erythrocyte lysis followed by negative immunomagnetic isolation using the Mouse Neutrophil negative isolation kit (Miltenyi, Germany). Cells were suspended in an RPMI based medium containing 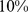 FCS and adjusted to a concentration of 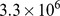 cells/ml. 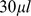 of this solution were added to the culture section of an Ibidi 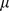
Slide VI 0.4 (Ibidi, Germany). Injected cells were allowed to settle for ten minutes in the incubator before both supplier wells were each filled with 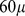
l medium. Then, cells visible in the culture section were imaged in a fully automated inverted cell culture microscope with environmental control (Leica, Germany) using a 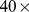 lens with conventional widefield illumination. The spatial resolution of the images was 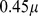
m/pixel and the resolution in time was one frame per 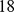 seconds. The migration of neutrophils was recorded (see Movie S1) for manual tracking based on the series of images.

The manual tracking of the cell was performed using the public domain open source software ImageJ [20] in combination with the MTrackJ plugin [2]. ImageJ requested about 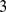 GB of RAM for the movie of 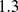 GB size. After movie import manual cell tracking was performed by highlighting the centroid of the cell under consideration and retrieving the x- and y-coordinates. The cell was subsequently followed through all frames of the movie and the procedure repeated for all cells. Neglecting cells that did not fully enter the field of view, the number of considered cell tracks was 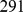. The number of time steps for these cell tracks ranged from 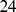 to 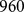 with average number 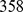 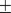 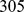. The accuracy in the manual determination of the cell centroid was checked by repeating the procedure for more than 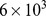 different cell positions. We found that the centroids of neutrophils, which have diameters in the range 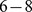 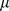
m, were accurately measured within an isotropic standard deviation of 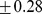 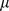
m.

## Results

We present the results on the automated characterization and parameter-free classification for synthetic cell tracks generated on the computer as well as for neutrophil migration observed in microscopy experiments.

### Cell population analyses obscure heterogeneity in cell track data

A statistical analysis was performed for a population of 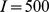 synthetic cell tracks that were generated *in silico* as outlined in the Methods section. The cell population was composed of three sub-populations each representing a distinct type of migration behavior with a different fraction of cell tracks: fairly straight cell tracks (type 1, see Fig. S1) with fraction 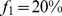, strongly confined cell tracks (type 2, see Fig. S2) with fraction 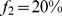, and purely random cell tracks (type 3, see Fig. S3) with fraction 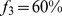.

**Figure 2:**
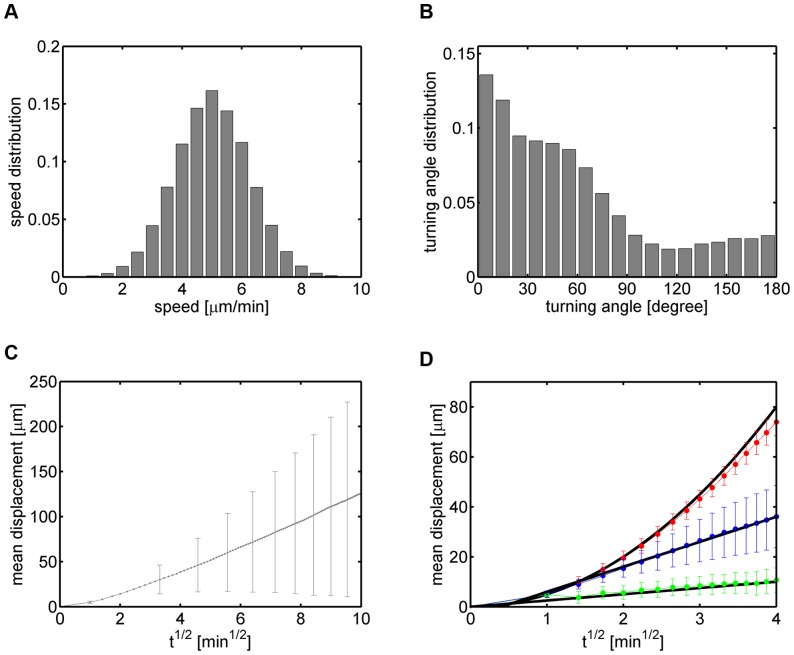
Cell population analyses of cell track data consisting of three sub-populations with distinct types of migration behavior. *(A)* Instantaneous speed distribution with average speed 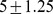 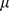
m/min. *(B)* Turning angle distribution with average angle 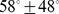. *(C)* Displacement curve showing linear dependence on the square-root of time for the overall cell population. Error bars correspond to the standard deviation and are only shown at selected time points to enhance clarity. *(D)* Displacement curves for each sub-population separately: 100 cell tracks of type 1 (red, see Fig. S1), 100 cell tracks of type 2 (green, see Fig. S2) and 300 cell tracks of type 3 (blue, see Fig. S3). Error bars correspond to the standard deviation.

In Fig. 2, we present the results of a cell population analysis. Based on the given cell tracks, we inferred the instantaneous speed distribution with average speed 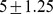 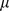
m/min, the turning angle distribution with average angle 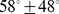 and the displacement curve. The latter was obtained by computing the vector 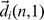 according to Eq. (9) for the 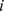
th cell and by averaging the length of the displacement vector over all cell tracks at time point 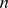. Of note, for a population of purely random cell tracks, 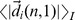 is expected to scale linear with the square-root of time 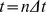,
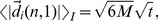
(18)where
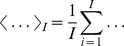
(19)refers to the population average and 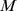 denotes the motility coefficient of the cells in three-dimensional space [21]. In contrast, for a population of cell tracks consisting of perfectly straight cell tracks only, 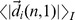 is expected to scale linear with time,
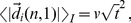
(20)assuming a constant cell speed 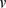.

Interestingly, even though 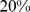 of all cell tracks in the cell population were of type 1 – *i.e.* corresponding to fairly straight cell tracks – the overall displacement curve still showed a linear scaling behavior (see Fig. 2C). We estimated the corresponding motility coefficient from the slope of the displacement curve to be 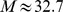 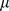
m
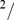
min at times 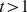 min. This example clearly demonstrates that a cell track analysis based on the cell population can be misleading, since the population's composition out of different sub-populations with distinct types of migration behavior is in general not known *a priori*.

In the present case of synthetic cell track data, the statistical analysis can as well be performed for each of the three sub-populations separately to demonstrate their differences in the scaling behavior with time (see Figs. S1–S3 and Fig. 2D). For the sub-population with cell tracks of type 1 the scaling behavior was found to be linear with time, *i.e.* quadratic in 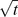, where the proportionality constant 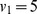 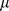
m
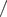
min was estimated from the average speed 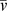 of all cells. Note that deviations from this scaling behavior at later time points reflect the fact that cell tracks of this population were chosen to be fairly but not perfectly straight and that the instantaneous speed was not constant but drawn from the distribution of speed values. On the other hand, in accordance with the underlying random migration behavior, the displacement for the sub-populations with cell tracks of type 2 and type 3 scaled linearly with the square-root of time and the corresponding motility coefficients were computed from the slopes of the displacement curves to be 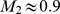 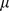
m
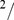
min and 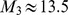 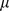
m
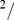
min, respectively. As expected, 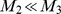, since type 2 cell tracks were much more strongly confined than type 3 cell tracks.

We conclude that the population analysis of cell track data does not only obscure relatively small heterogeneities in the cell track data, *e.g.* as was suggested for germinal center B cells in the presence of weak and transient chemotaxis [7], but even shows severe shortcomings in cases where the composition of the cell track population does have a significant heterogeneity over different sub-populations. The difference between sub-populations with characteristic migration behavior can not be resolved at the level of cell population analyses.

### Linear measures yield poor characterization of cell migration

**Figure 3:**
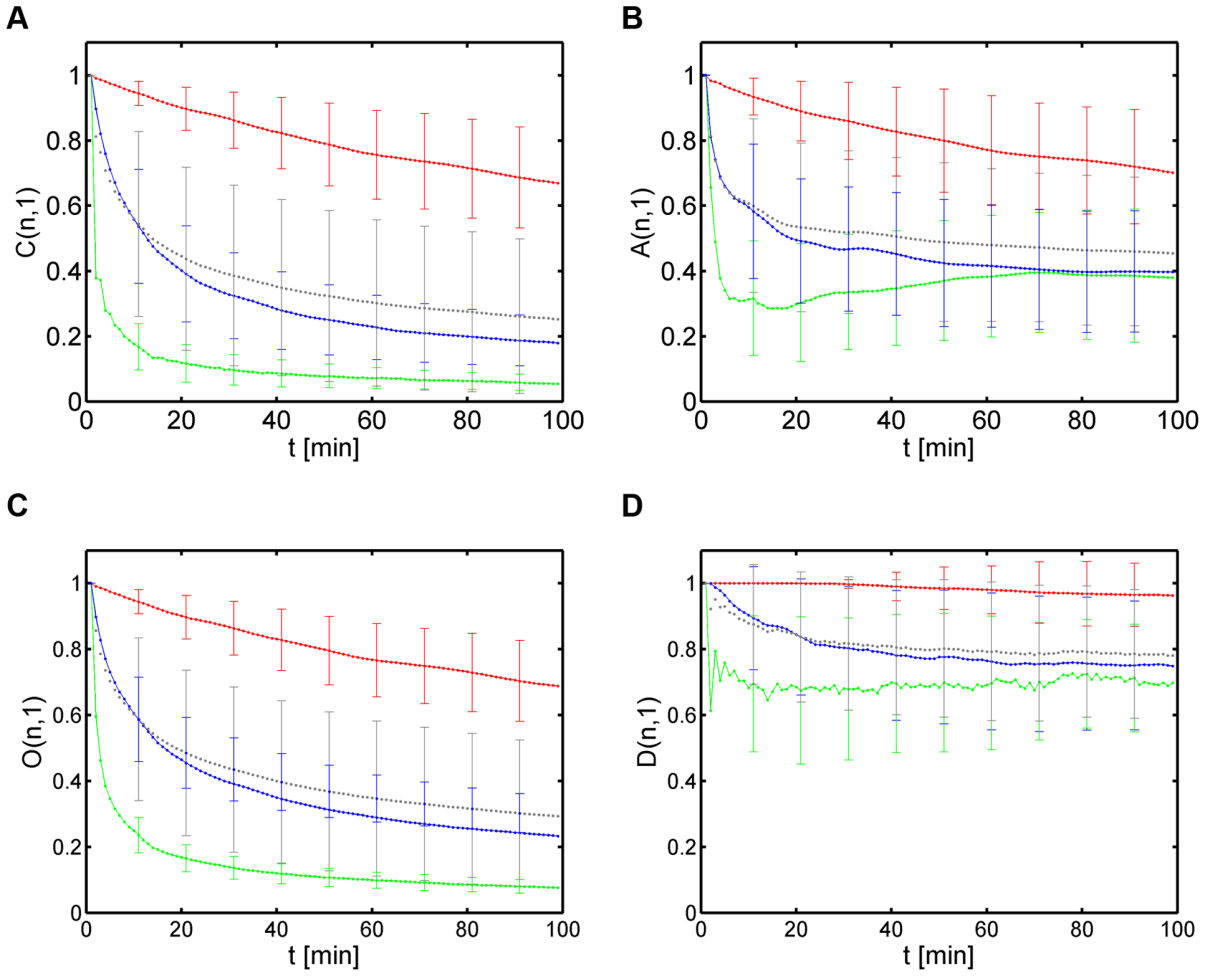
Characterization of cell populations by linear measures. Results are presented as time-dependent averages over the relevant cell populations for each type of migration behavior (type 1: red curve, type 2: green curve, type 3: blue curve) and for the overall population (grey curve). Error bars correspond to the standard deviation and are only shown at selected time points to enhance clarity. *(A)* Confinement ratio. *(B)* Volume asphericity. *(C)* Outreach ratio. *(D)* Displacement ratio.

The analysis of cell tracks can be extended to the calculation of various measures, such as volume asphericity 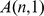, volume prolateness 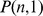, confinement ratio 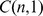, displacement ratio 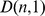 and outreach ratio 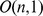 (see Methods section and Fig. 1). We refer to these measures as *linear measures*, since they were computed at time point 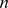 along the cell track relative to the initial position of the cell (at time point 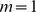). The results are shown in Fig. 3 for the population of all synthetic cell tracks and for each sub-population separately.

It is instructive to briefly discuss the confinement ratio
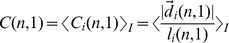
(21)which is obtained for the 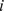
th cell track as the ratio of the length of the direct path 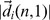 between the initial and the current position 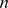 over its current track length 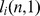 and by taking the population average Eq. (19) over all relevant cell tracks. A perfectly straight cell track is characterized by 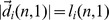 with confinement ratio 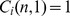, indicating that the track is not confined at all. In contrast, a cell that does not migrate along a perfectly straight track has a path length that is always larger than the length of its displacement vector, 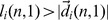, and, thus, 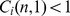 for 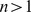. Assuming, for the sake of simplicity, that the instantaneous cell speed 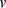 is constant, the length of a cell track is linearly scaling with time, 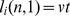, irrespective of the type of migration behavior. For a population of cells performing random walk migration the length of the average displacement vector scales according to Eq. (18) with the square-root of time, such that the average confinement ratio behaves as 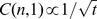. In principle, after a sufficiently large number of time steps 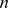, the confinement approaches zero: 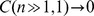. If the population of cell tracks consists of different sub-populations with distinct types of migration behavior or if single cell tracks consist of a mixture of random and straight segments, the convergence of 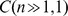 can be slow, depending on the relative contributions from random and straight migration. This can give rise to ambiguous situations, as was observed for the confinement ratios plotted in Fig. 3A, where the grey curve refers to the overall cell population and can hardly be distinguished from the sub-population of cell tracks with pure random walk migration (type 3, blue curve).

It can be concluded that the confinement ratio 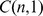 provides only a poor characterization of cell migration that may not suffice to infer distinct sub-populations for different types of migration behavior. The same statement holds for the other linear measures, as can be seen in Fig. 3B–D. In case of the volume asphericity 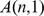, type 2 and type 3 migration can not be distinguished at large time points 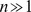. This is a direct consequence of the fact that the number of cell tracks, which are upto a scaling factor compatible with one and the same track volume, increases with the number of time points 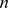 like 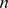-factorial (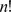). Therefore, populations of type 2 and type 3 can become comparable with regard to the value of the volume asphericity for 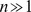. Furthermore, 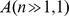 approaches a finite value indicating that the cell track topology is not spherical, which is a consequence of the fact that the underlying distributions for the turning angle and the speed are not homogeneous in both cases (see Figs. S2 and S3).

In passing we note that the time-dependent behavior of the volume prolateness 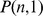 was generally observed to be very similar to the volume asphericity (see Fig. S4A for comparison with Fig. 3B). This similarity is expected for cell tracks with prolate topology, *i.e.* associated with cigar-shaped ellipsoidal volumes, which is in agreement with the above considerations on the volume asphericity alone. Since in any case the application of volume prolateness is limited to cell tracks in three spatial dimensions, we did not consider this measure in what follows to keep the subsequent analysis most general. However, this measure may contribute additional information in the rare event of a cell population that is characterized by cell tracks with oblate topology in three spatial dimensions.

Next, the outreach ratio 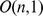 in Fig. 3C shows a time-dependent behavior that is similar to that of the confinement ratio. While this was expected for fairly straight cell tracks where the maximal displacement equals the current displacement of the cell, this was also found for the two populations with cell tracks of type 2 and type 3. Once again, this reflects the prolate topology of these cell tracks and since this characteristic behavior can always be expected for non-homogeneous distributions of speed and turning angle, the discrimination between cell tracks of type 2 and type 3 by 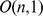 is as unpromising as by 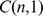. Finally, the displacement ratio 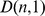 in Fig. 3D showed an overall shift to higher values but beyond that no characteristic features could be identified that would provide direct information on the composition of the cell population.

In summary, linear measures of cell populations are altogether lacking the sensitivity required for an unambiguous discrimination of different types of migration behavior at the level of a population of cells.

### Staggered measures are sensitive to local migration behavior

**Figure 4:**
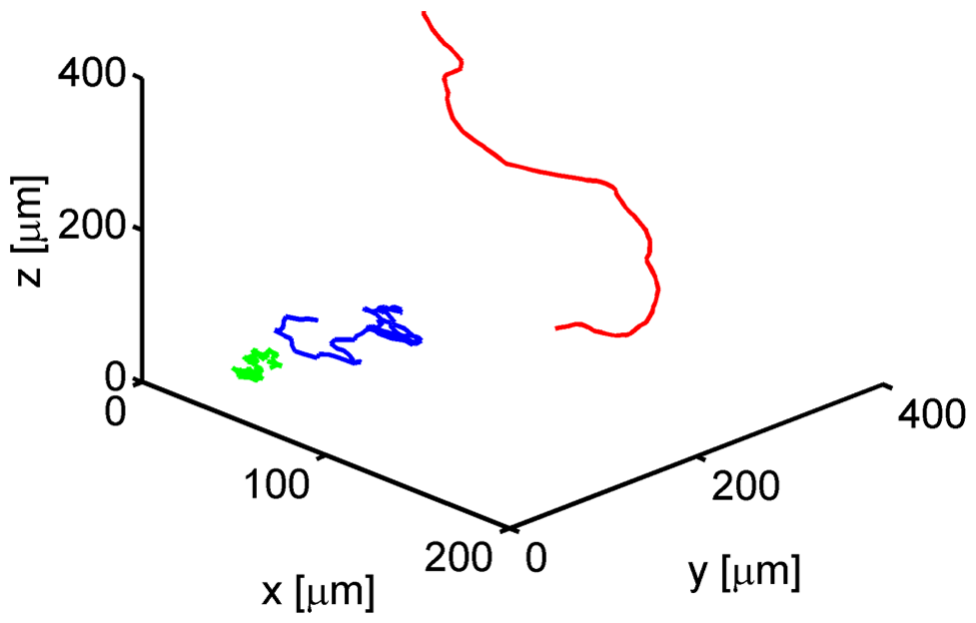
Examples of cell tracks from different sub-populations. One cell track for each type of migration behavior is shown: a fairly straight cell track (type 1, red), a strongly confined cell track (type 2, green) and a purely random cell track (type 3, blue).

**Figure 5:**
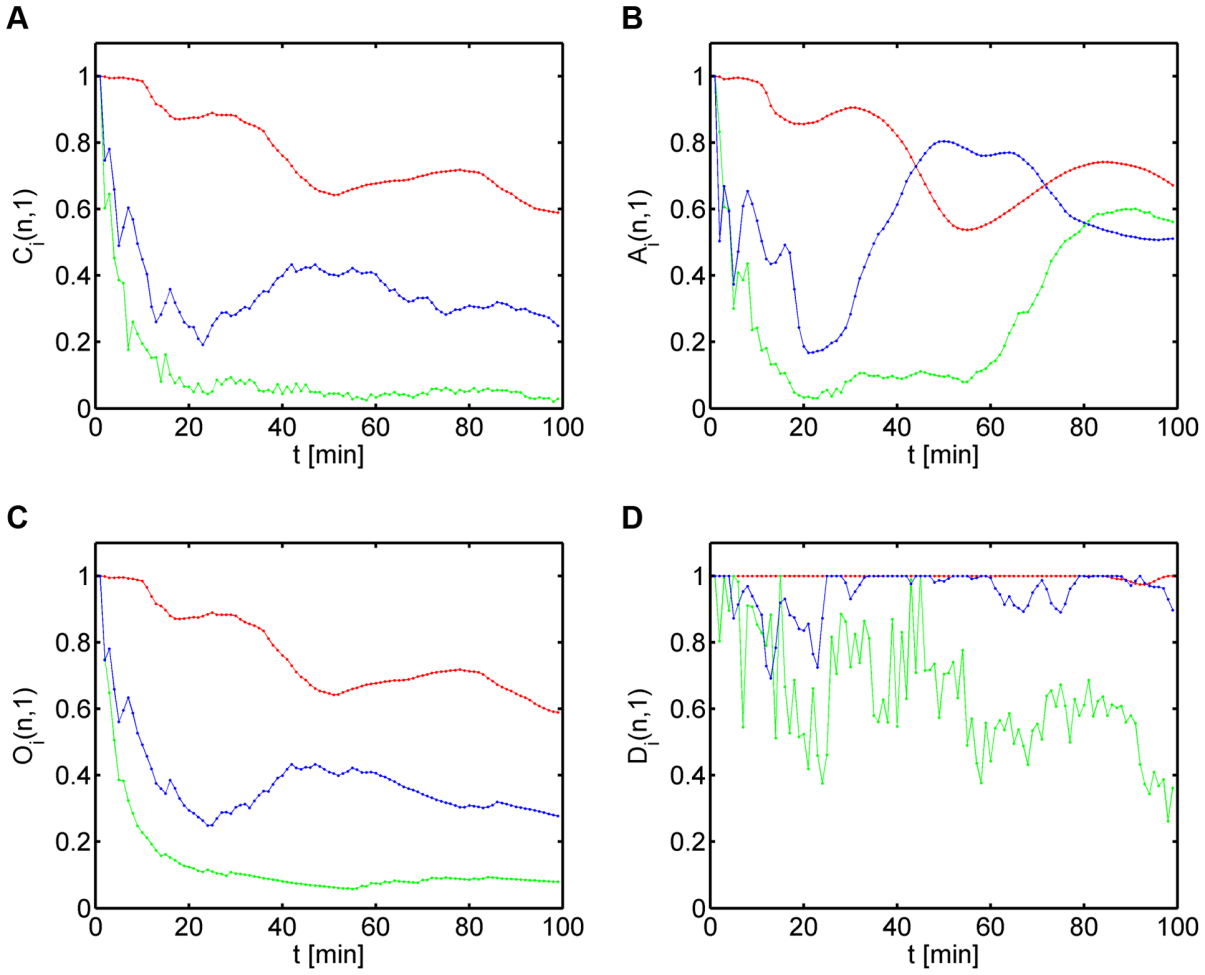
Linear measures for the three types of cell tracks shown in Fig.(type 1: red, type 2: green, type 3: blue). *(A)* Confinement ratio as a function of time. *(B)* Volume asphericity as a function of time. *(C)* Outreach ratio as a function of time. *(D)* Displacement ratio as a function of time.

Analyses at the population level yield only a poor characterization of cell migration and, thus, do not provide the information required to decompose a cell population into different sub-populations with distinct migration behavior. We therefore extended the above considerations by computing the linear measures for single cell tracks in order to identify characteristic signatures that could be related to their migration behavior. In Fig. 4, we plot three synthetic cell tracks that were chosen as representatives from the three sub-populations. The corresponding linear measures 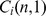, 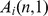, 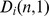 and 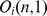 for the 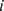
th cell track are summarized in Fig. 5, while for the volume prolateness 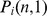 we refer to Fig. S4B.

We found that, as expected, linear measures of single cell tracks convey more detailed information on the migration behavior. For example, focusing on the cell track of type 3 migration (blue curves in Fig. 5), the confinement ratio still scaled like 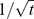 on average. However, temporal phases with increasing values of 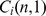 corresponded to track segments that were relatively straight (see Fig. 5A). A clear identification of these transient phases was hindered by short-term fluctuations in 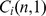, however, we generally observed these to be reduced for the outreach ratio 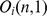 (see Fig. 5C). Correspondingly, in the transient regions of increasing track straightness, the volume asphericity 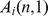 increased as well (see Fig. 5B), since the overall track volume progressively deviated from a sphere and became more prolate (see Fig. S4B for comparison with 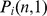). A similar observation was made for the displacement ratio 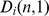 (see Fig. 5D), which reached high values in the transient regions of increasing track straightness.

While linear measures of single cell tracks generally convey more detailed information, they still do suffer from the burden that quantities at time point 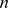 are computed as the average over all previous time points 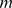 with 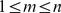. This implies that identical track segments at different positions along the cell track give rise to different impact on the linear measures, since the track segment at the later time point enters the averaging relative to the initial time point with less weight. Therefore, with increasing number of time points linear measures lose sensitivity for temporal changes in the cell migration behavior.

To capture the local migration behavior we extended the computation of linear measures to the *staggered measures* 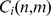, 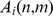, 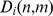 and 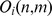. In this case, each point 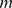 along the cell track was considered as the initial point of the cell track, *i.e.* linear measures were separately computed relative to each previous time point along the cell track segment with 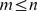. As was shown in the Methods section, each staggered measure corresponds to a symmetric matrix with entries varying between 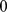 and 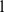. These matrices can be represented by heat maps with the common property that the value along the diagonal is always 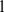, since 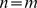 involves one migration step which always is a straight step by construction.

**Figure 6:**
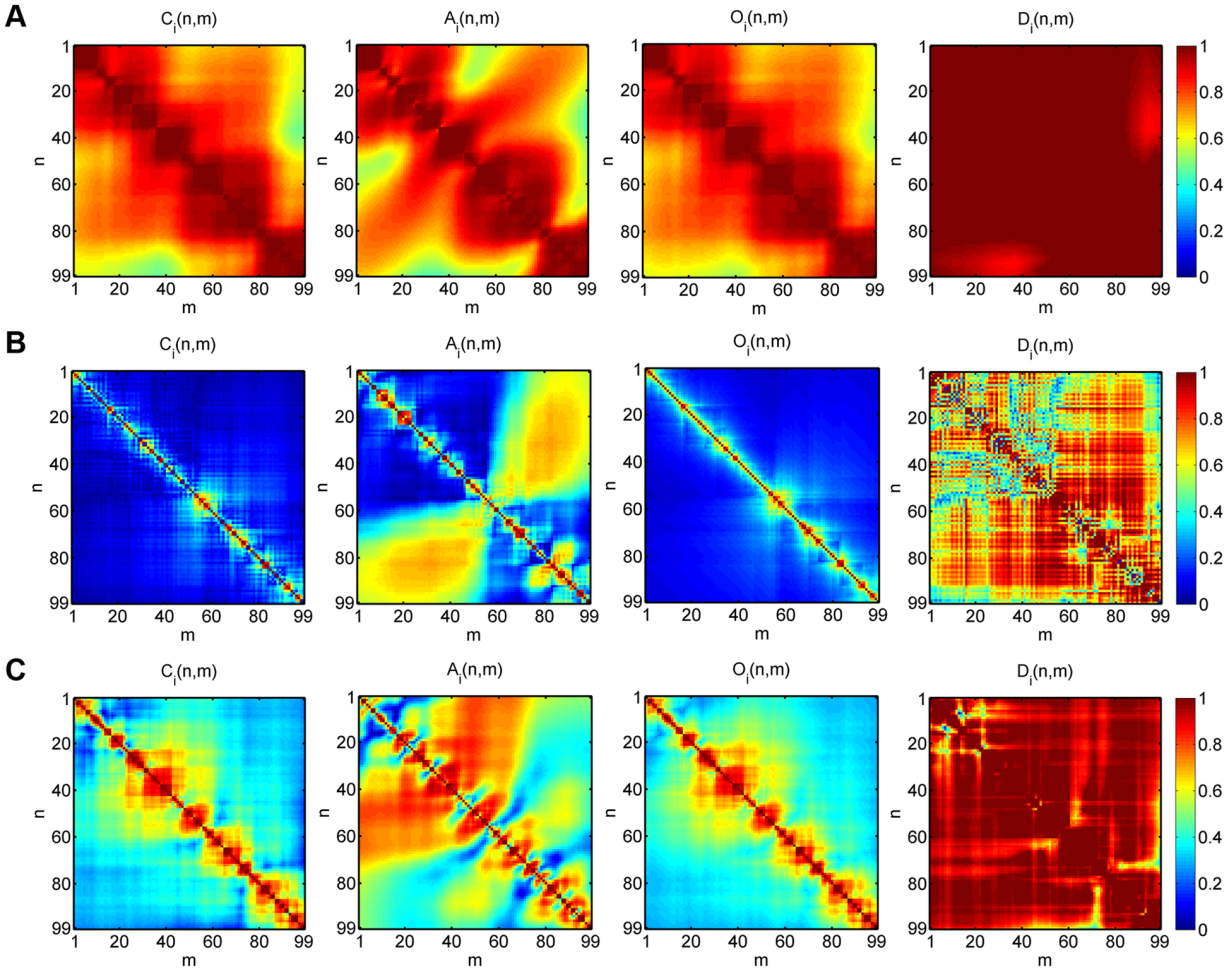
Heat maps of the staggered confinement ratio, staggered volume asphericity, staggered outreach ratio and staggered displacement ratio for the three types of cell tracks shown in Fig. 4. *(A)* Type 1: fairly straight cell track. *(B)* Type 2: strongly confined cell track. *(C)* Type 3: purely random cell track.

In Fig. 6, we plot the heat maps of the staggered measures for the three representative cell tracks presented in Fig. 4, while for the volume prolateness 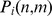 we refer to Fig. S5. The first column of a heat map is identical to the linear measure along the cell track relative to the initial position of the cell track and was presented in Fig. 5. Going along the diagonal of the matrix corresponds to advancing the initial position of the cell track in the calculation of the linear measure that is again shown downwards along the corresponding column. The staggered measures contain detailed information on the local migration behavior of the cell. For example, large values of the staggered measures correspond to straight track segments and their spatial distribution in the biological sample can be directly inferred via the matrix from the occurrence of the corresponding temporal phase (see Eq. (3)).

The advantage of computing staggered measures with regard to the characterization of cell migration is obvious: identical track segments at different positions along the cell track give rise to the same impact on the linear measures, since the track segment at the later time point enters the averaging relative to its shifted initial time point with the same weight. For example, in the case of the type 3 cell track (see Fig. 6C) the heat map for the confinement ratio contains several extended regions centered along the diagonal with large values that are indicative for straight track segments. In the first column of the heat map, which corresponds to the linear measure 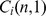 in Fig. 5A (blue curve), these regions only appear as shifted and smeared out regions being less pronounced at later time points. Similarly, the volume asphericity 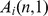 (see blue curve in Fig. 5B) can be misleading because local changes in 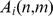 (see Fig. 6C) get averaged out: with increasing number of time points 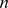 the degeneracy of the ellipsoidal volume is increasing like 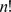 reflecting the loss of information at the local scale. In other words, large values in 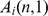 can be maintained indicating fairly straight migration even though the values 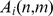 at the local scale are small because strongly confined track segments exist.

We conclude that the inference of the spatial accumulation of straight track segments can be achieved on the basis of staggered measures that accurately capture the local migration behavior of cells. The analysis of heat maps for the staggered measures is straightforward and readily allows identifying the spatial distribution of specific migration types in the biological sample by the unique relation between time points and absolute positions in the sample.

### Hierarchical clustering reveals heterogeneity in cell track data

Exploiting the fact that staggered measures of cell tracks are sensitive to the local migration behavior, we considered the possibility to annotate cell track data by the average values of the staggered measures, *e.g.* we computed the average staggered confinement ratio from the corresponding matrix by
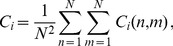
(22)and similar for the other staggered measures. It should be noted that averaged staggered measures are strongly biased by the transient migration behavior of the cell tracks, since the average of the linear measure relative to the initial cell position, 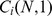, enters the averaging procedure only with weight 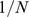. This implies that averaging staggered measures conveys local information and the results for the three representative cell tracks shown in Fig. 4 are summarized in Table 1. We found for each average staggered measure that the largest and smallest values are obtained, respectively, for fairly straight cell tracks (type 1) and strongly confined cell tracks (type 2). Intermediate values for the average staggered measures were attributed to type 3 cell tracks performing random walk migration.

**Table 1:** Average staggered measures of three representative synthetic cell tracks per migration type.

average staggered	type 1:	type 2:	type 3:
measure	fairly straight	strongly confined	purely random
confinement ratio	0.81 (0.86 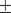 0.05)	0.14 (0.13 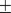 0.02)	0.45 (0.39 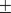 0.05)
volume asphericity	0.80 (0.86 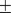 0.06)	0.39 (0.34 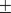 0.08)	0.57 (0.50 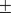 0.07)
outreach ratio	0.82 (0.86 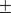 0.05)	0.19 (0.18 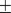 0.02)	0.48 (0.44 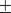 0.04)
displacement ratio	0.99 (0.99 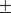 0.02)	0.69 (0.71 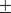 0.06)	0.93 (0.83 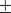 0.06)
volume prolateness	0.74 (0.81 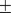 0.08)	0.33 (0.28 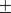 0.06)	0.48 (0.41 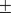 0.07)

For each average staggered measure the values are highest (lowest) in the case of cell tracks of type 1 (type 2), while cell tracks of type 3 always assume intermediate values. Values in brackets denote the average value and standard deviation for the average staggered measure of the corresponding sub-population. The Wilcoxon rank-sum test revealed that the average staggered measures of different sub-populations were significantly different (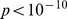).

**Figure 7:**
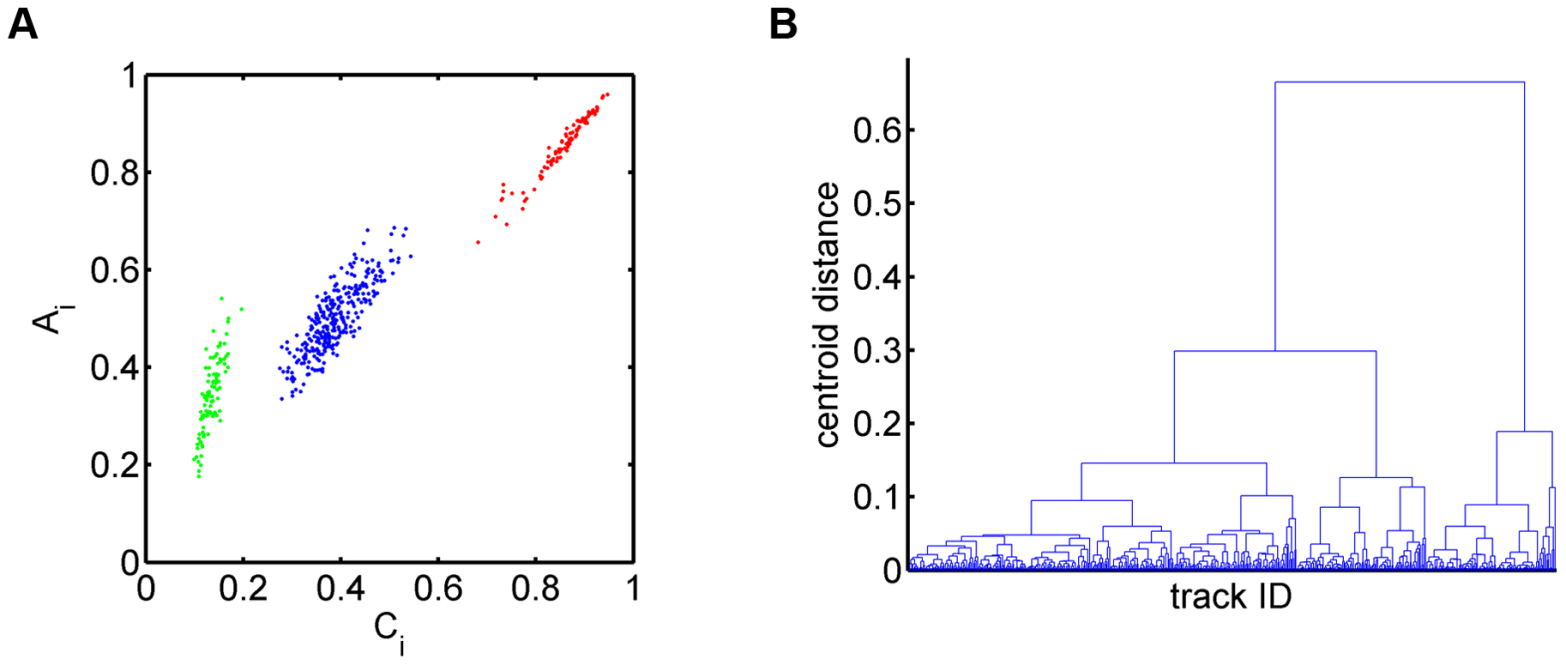
Hierarchical clustering of synthetic cell tracks in the parameter space of staggered measures. *(A)* Cell tracks from the three sub-populations with different types of migration behavior, *i.e.* fairly straight (type 1: red), strongly confined (type 2: green) and purely random (type 3: blue), form distinct clusters in the space spanned by the average confinement ratio and the average volume asphericity. *(B)* Dendrogram obtained from the agglomerative hierarchical clustering based on the euclidean distance between the centroids of groups of data points.

These findings led us to consider average staggered measures as the basis of a parameter space where cell tracks can be clustered by their migration behavior. In fact, a plot of the population of 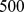 synthetic cell tracks in the parameter space defined by 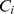 and 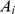 is shown in Fig. 7A and readily reveals three distinct clusters referring to type 1 (red), type 2 (green) and type 3 (blue) cell tracks. Applying the method of agglomerative hierarchical clustering using the Euclidean distance metric with regard to the centroid position of groups of data points (see Methods section), we obtained the dendrogram presented in Fig 7B. As expected, the three clusters inferred from the dendrogram correspond to the clusters for the three types of migration in Fig. 7A.

Of note, the identified clusters of sub-populations could not be obtained in the parameter space of average linear measures. For example, we computed the average confinement ratio of the linear measure,
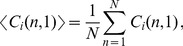
(23)and correspondingly for the linear volume asphericity. In close analogy to Fig. 7 for the average staggered measures, we computed the average linear measures for each cell track and represented the data points in the parameter space of average linear measures (see Fig. S6A). We found that clustering in this parameter space did not recover the *a priori* known sub-populations with different types of migration behavior. In Fig. S6B we plotted the three sub-populations predicted from clustering based on average linear measures in the parameter space of average staggered measures for direct comparison with Fig. 7A. To quantify this observation, we determined the true positives (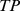), false positives (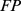), true negatives (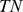) and false negatives (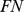) for each of the three sub-populations in Fig. S6B and then computed the corresponding accuracy 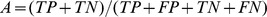 of the classification (see Table S1 for details). We obtained 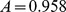 for fairly straight cell tracks (type 1), 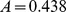 for strongly confined cell tracks (type 2) and 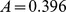 for purely random cell tracks (type 3). This gives rise to an accuracy of 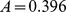 for the overall classification and these values should be contrasted with those from clustering based on average staggered measures that always yield the maximal value 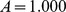 for each migration type separately as well as for the overall classification. This shows the importance of local information contained in the staggered measures for the clustering of cell tracks.

**Figure 8:**
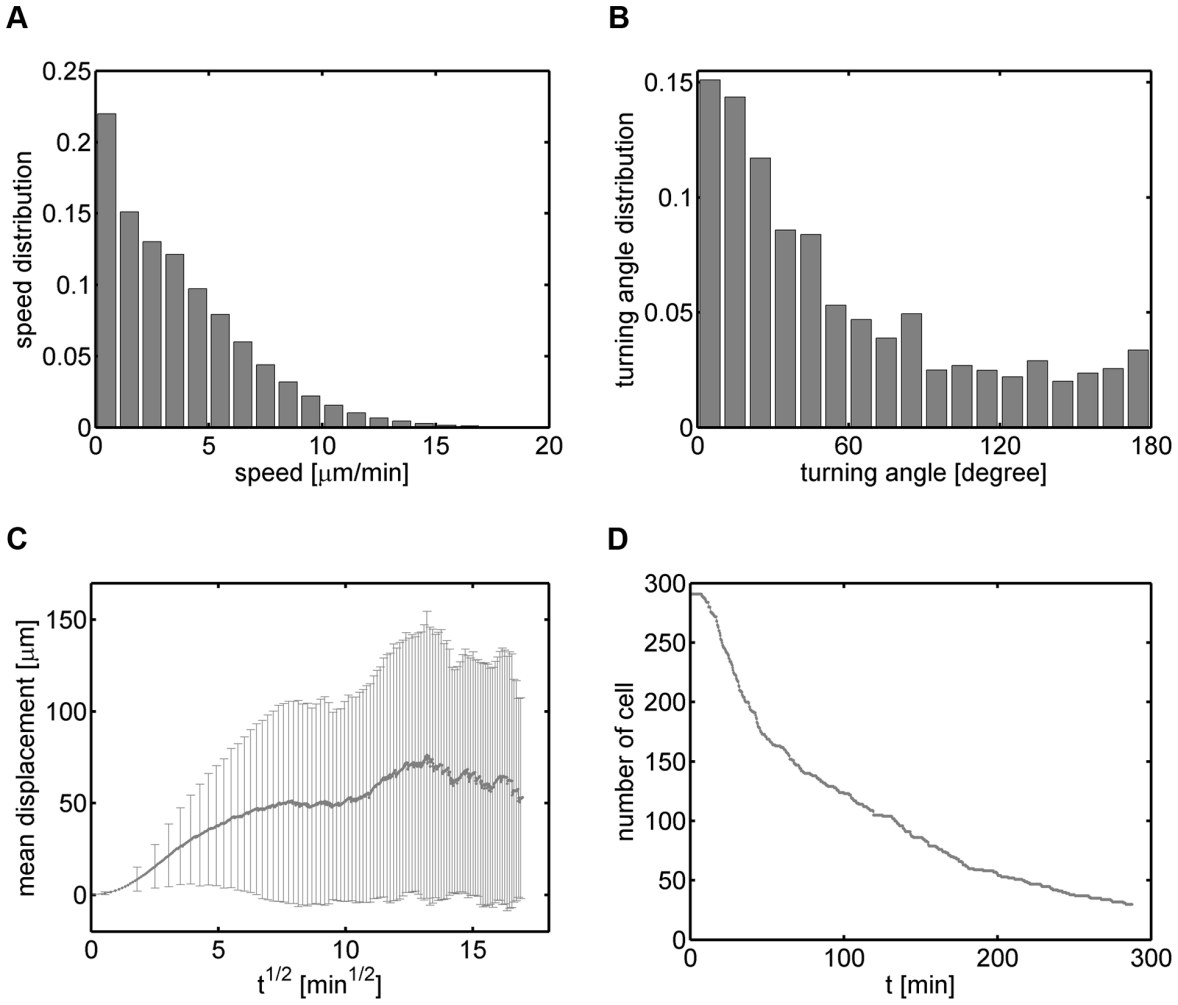
Cell population analyses of cell track data obtained from neutrophil migration. *(A)* Instantaneous speed distribution with average speed 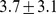 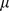
m/min. *(B)* Turning angle distribution with average angle 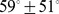. *(C)* Displacement curve showing linear dependence on the square-root of time. Error bars correspond to the standard deviation and are only shown at selected time points to enhance clarity. *(D)* Number of cell tracks as a function of time.

Going beyond the analyses of synthetic cell tracks, we applied the automated characterization and parameter-free classification also to real cell track data, which were obtained by manually tracking neutrophils that we observed by *in vitro* microscopy experiments in two spatial dimensions. In Fig. 8 we plot the results of a cell population analysis in terms of the speed distribution with average speed 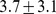 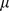
m/min, turning angle distribution with average angle 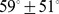 and the average displacement as a function of the square-root of time. Note that a total number of 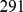 cells was tracked up to roughly 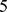 hours (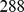 minutes) with an average track duration of nearly 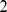 hours (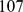 minutes). Of course, in the present case, nothing is known *a priori* about the existence of sub-populations.

Due to the rich diversity of cell tracks in general, their classification into different types of migration behavior can be a challenging task. In contrast to the data set of synthetic cell tracks, it can be expected that experimental data points in the parameter space of the average staggered measures are not organized in clearly distinct clusters. Reasons for these effects being correlations between the speed and the turning angle in cellular migration or different lengths of experimental cell tracks following a non-uniform distribution. The latter impact may be reduced by restricting the analysis to cell tracks with a minimum number of time points. Therefore, describing sub-populations in picturesque terms like *fairly straight*, *strongly confined* and *purely random* should be taken with some care and understood as, respectively, combinations of relatively high, low, and intermediate values in the average staggered measures. In addition, clustering results may depend on the combination of the four average staggered measures that give rise to eleven possible parameter spaces, *i.e.* six and four combinations for clustering in a two-dimensional (2D) and three-dimensional (3D) parameter space, respectively, and one parameter space for 4D clustering that combines all four average staggered measures.

**Figure 9:**
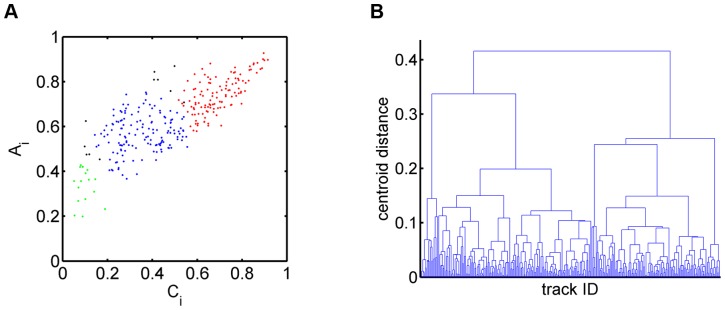
Hierarchical clustering of neutrophil cell tracks in the parameter space of two staggered measures. *(A)* Sub-populations of cell tracks in the space spanned by the average confinement ratio and the average volume asphericity: fairly straight cell tracks (type 1: red), strongly confined cell tracks (type 2: green) and purely random cell tracks (type 3: blue). In going from 2D to 4D clustering, eleven cell tracks change sup-populations from type 1 to type 3 and from type 3 to type 2 (indicated in black). *(B)* Dendrogram obtained from the agglomerative hierarchical clustering based on the euclidean distance between the centroids of groups of data points.

The result of 2D clustering is shown in Fig. 9 where the average staggered confinement ratio and the average staggered volume asphericity form the basis of the parameter space. In contrast to the case of synthetic cell tracks (see Fig. 7), neutrophil cell tracks were found to be much more spread out and a clear distinction between clusters of cell track types was not apparent. Nevertheless, the three clusters inferred from the dendogram of hierarchical clustering are colored in red, green and blue in Fig. 9A and were found to be clearly distinct in their migration behavior. This was determined by computing the average and standard deviation in the staggered measures for each cluster (see Tables 2–4 and Fig. S7). Performing statistical tests as outlined in the Methods section revealed that the average staggered measures of different sub-populations were significantly different (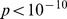). Furthermore, we also checked the cluster size because small clusters may contain only a few outliers (see Tables 2–4 and Fig. S8). Note that cell tracks of type 1 strongly differ in the average length of cell tracks compared with type 2 and type 3 cell tracks. This is a direct consequence of the limited field of view that is traversed by these relatively straight cell tracks, as can be deduced from the correlation with the average number of time steps (see Tables 2–4). For the same reasons, the highest average number of time steps is found for cell tracks of type 2 because strongly confined cell tracks were monitored during a large fraction of the recording time.

**Figure 10:**
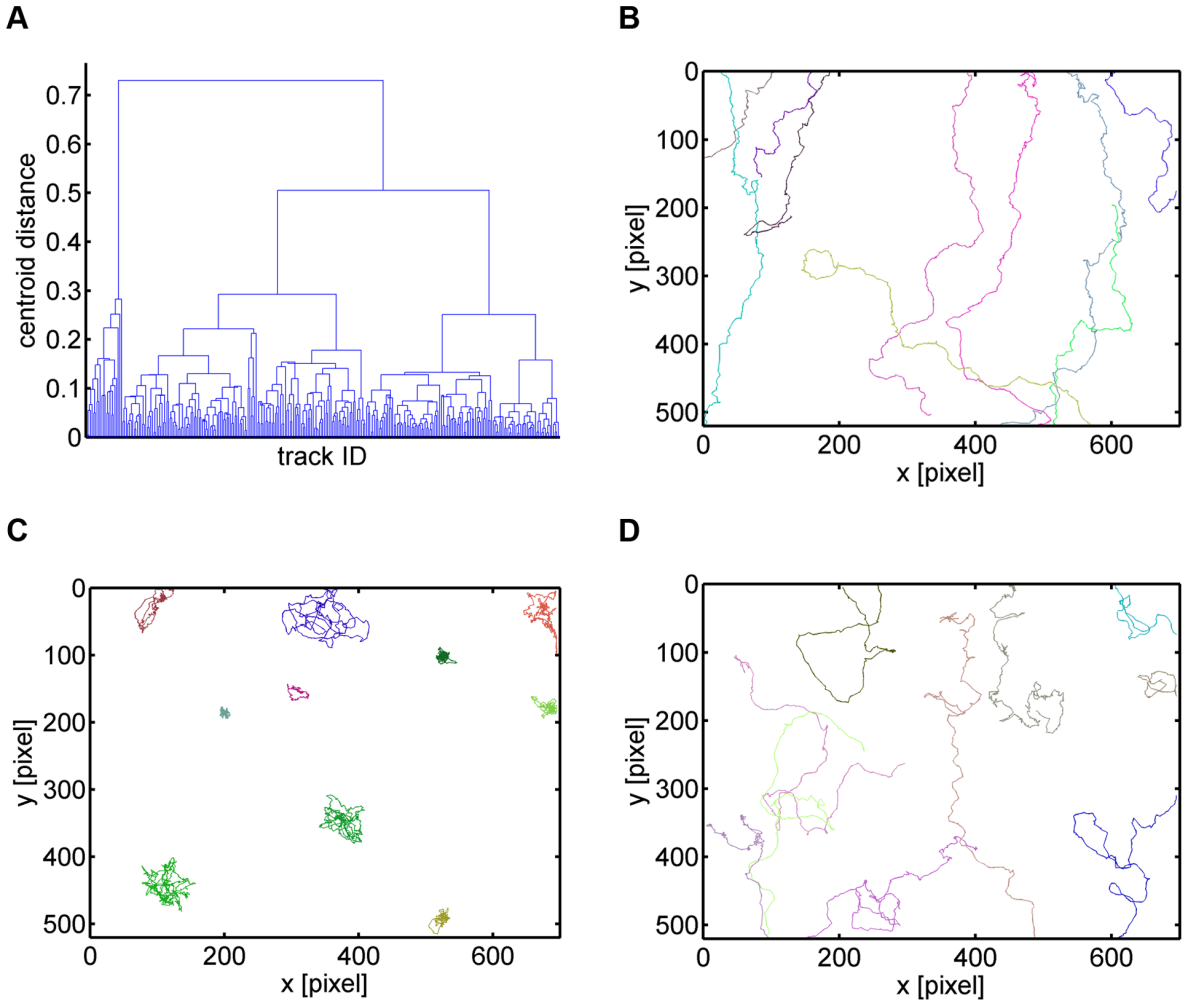
Hierarchical clustering of neutrophil cell tracks in the parameter space of four staggered measures. *(A)* Dendrogram obtained from the agglomerative hierarchical clustering based on the euclidean distance between the centroids of groups of data points. The clustering was performed in the space spanned by the average confinement ratio, average volume asphericity, average displacement ratio and average outreach ratio. *(B)* Representative cell tracks of the cluster with fairly straight cell tracks. *(C)* Representative cell tracks of the cluster with strongly confined cell tracks. *(D)* Representative cell tracks of the cluster with purely random cell tracks.

Changing the dimension of the parameter space to 4D clustering based on all four average staggered measures revealed that the migration type of only eleven out of 291 cell tracks – *i.e.* less than 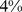 – was affected (see Tables 2 and 4 and Fig. S8): six cell tracks changed sub-populations from type 1 to type 3 and five cell tracks changed sub-populations from type 3 to type 2. All other cell tracks were classified to the same sub-populations as obtained from 2D clustering with the average staggered confinement ratio and the average staggered volume asphericity. The dendrogram of 4D clustering together with representative cell tracks per migration type are presented in Fig. 10.

Based on 4D clustering, a cell population analysis was performed for each sub-population separately (see Figs. S9–S11). We found that the average speed and average turning angle of type 1 cell tracks is given by, respectively, 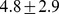 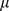
m/min and 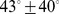 and the displacement curve scales with 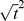 during the first few minutes. In contrast, cell tracks of type 2 (type 3) have average speed 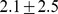 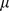
m/min (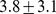 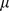
m/min) and average turning angle 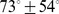 (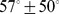) with displacement curves scaling roughly linear with 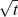. The corresponding motility coefficients were inferred from the linear slopes to be 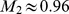 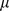
m
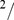
min for type 2 and 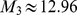 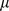
m
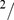
min for type 3 cell tracks.

We stress again that the identified clusters of sub-populations could not be obtained in the parameter space of average linear measures. This is shown in Fig. S12, where we plotted the parameter space of average linear measures using red, green and blue color according to the cell migration types 1, 2, and 3, respectively, that were previously obtained from the clustering in the parameter space of average staggered measures (see Fig. 9). The data points appeared to be strongly intermixed, indicating – as expected from the above considerations for synthetic cell tracks – that identification of sub-populations with the same migration behavior is not possible in the parameter space of average linear measures.

In summary, we demonstrated that hierarchical clustering can be successfully applied to identify sub-populations of different migration types contained in a population of real cell track data. The resulting sub-populations were found to be quantitatively robust against changing dimensions of the parameter space from 2D clustering based on the average staggered confinement ratio and the average staggered volume asphericity to 4D clustering based on all four average staggered measures.

### Combination of staggered measures improves classification results

Our result regarding the quantitative robustness of the identified sub-populations with regard to 2D and 4D clustering was further investigated by comparing all eleven combinations of the four staggered measures for 2D clustering (six combinations abbreviated by CA, CO, CD, AO, AD and OD), 3D clustering (four combinations abbreviated by CAO, CAD, COD and AOD) and 4D clustering (one combination abbreviated by CAOD). This detailed analysis was motivated by the fact that the eleven switching cell tracks in the CAOD clustering in 4D were not always located at the borders of their sub-populations in CA clustering in 2D, as indicated in Fig. 9A in black color. Thus, even though switching upon changing the dimension of the parameter space was exclusively observed between clusters that were direct neighbors, it obviously was associated with more drastic changes in the sub-populations than just a fine-tuning at the borders between neighboring clusters.

The analysis revealed that the combination of average staggered measures strongly influences the classification result. A quantitative summary of the analysis for all eleven combinations of 2D, 3D and 4D clustering is presented in Tables 2–4 and plotted in Fig. S7 for the average staggered measures and in Fig. S8 for the number of cell tracks per sub-population. The main finding is that the combination of the staggered confinement ratio and the staggered volume asphericity plays the key role in the meaningful clustering of cell tracks (CA clustering). This can be explained by their opposing view on cell track segments, because a small value in the average staggered confinement ratio may still be balanced by an increased value in the average staggered volume asphericity for a globally elongated cell track that is locally coiled. In contrast, since the average staggered confinement ratio and the average staggered outreach ratio characterize cell tracks in a similar fashion, it is not surprising that the combination of these two quantities alone yielded unreasonable classification results (CO clustering). This interpretation is confirmed by the observation that replacing the average staggered confinement ratio by the average staggered volume asphericity did again yield quantitatively comparable results in combination with the average staggered outreach ratio (AO clustering).

Following this line of argumentation, it can be concluded that the average staggered displacement ratio in two spatial dimensions is more related to the average staggered volume asphericity than to any other measure. Therefore, clustering based on these two measures yielded unreasonable results (see Table 2 for AD clustering). Interestingly, we observed that the average staggered displacement ratio behaves differently in combination with the average staggered confinement ratio and the average staggered outreach ratio (compare CD and OD clustering in Fig. S7).

**Figure 11:**
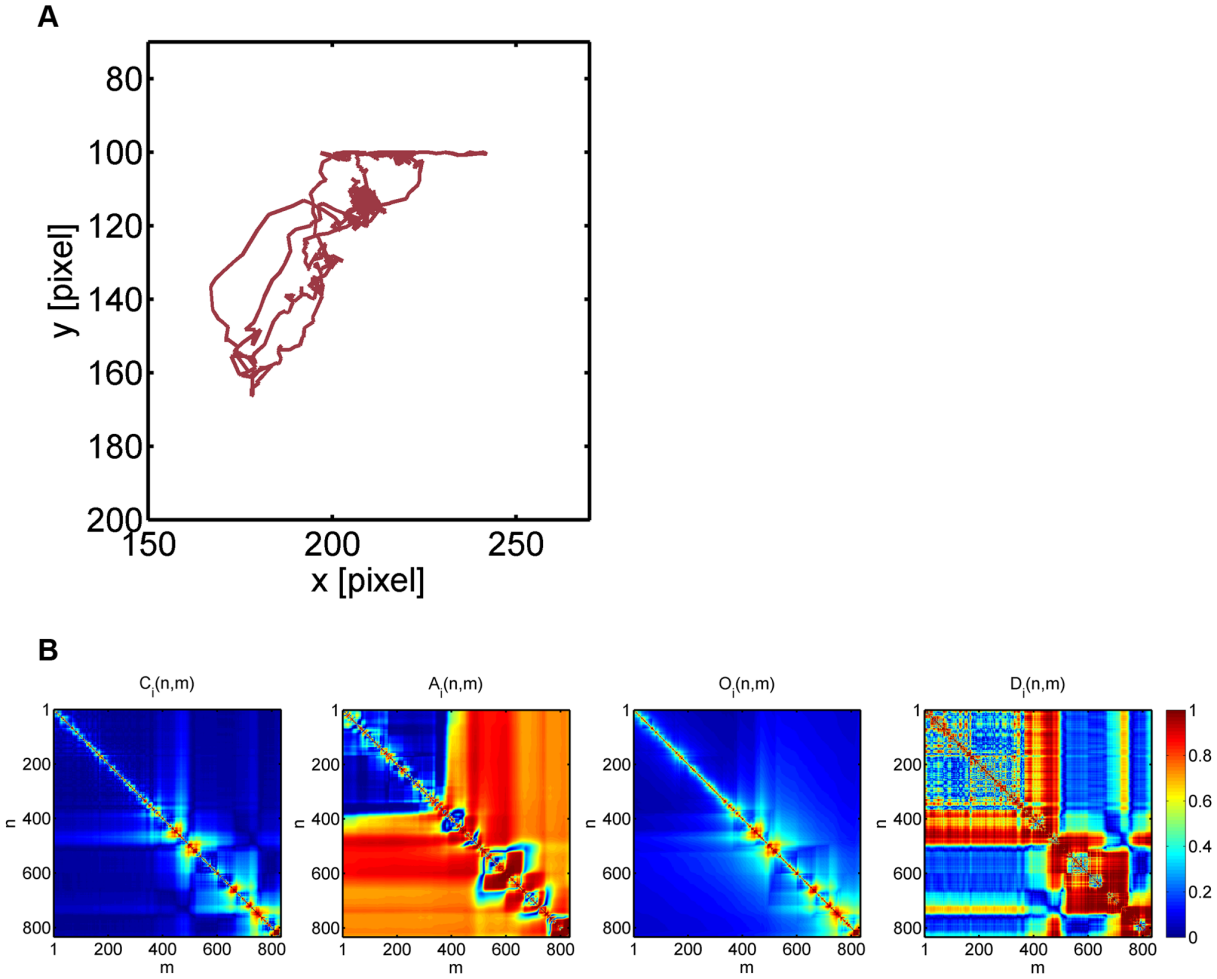
Neutrophil cell track with ID 1 switching sub-populations in going from 2D to 4D clustering. *(A)* Neutrophil with track ID 1 switched from sub-population of type 3 (random migration) to type 2 (strongly confined migration). *(B)* Heat maps of the staggered confinement ratio, staggered volume asphericity, staggered outreach ratio and staggered displacement ratio.

**Figure 12:**
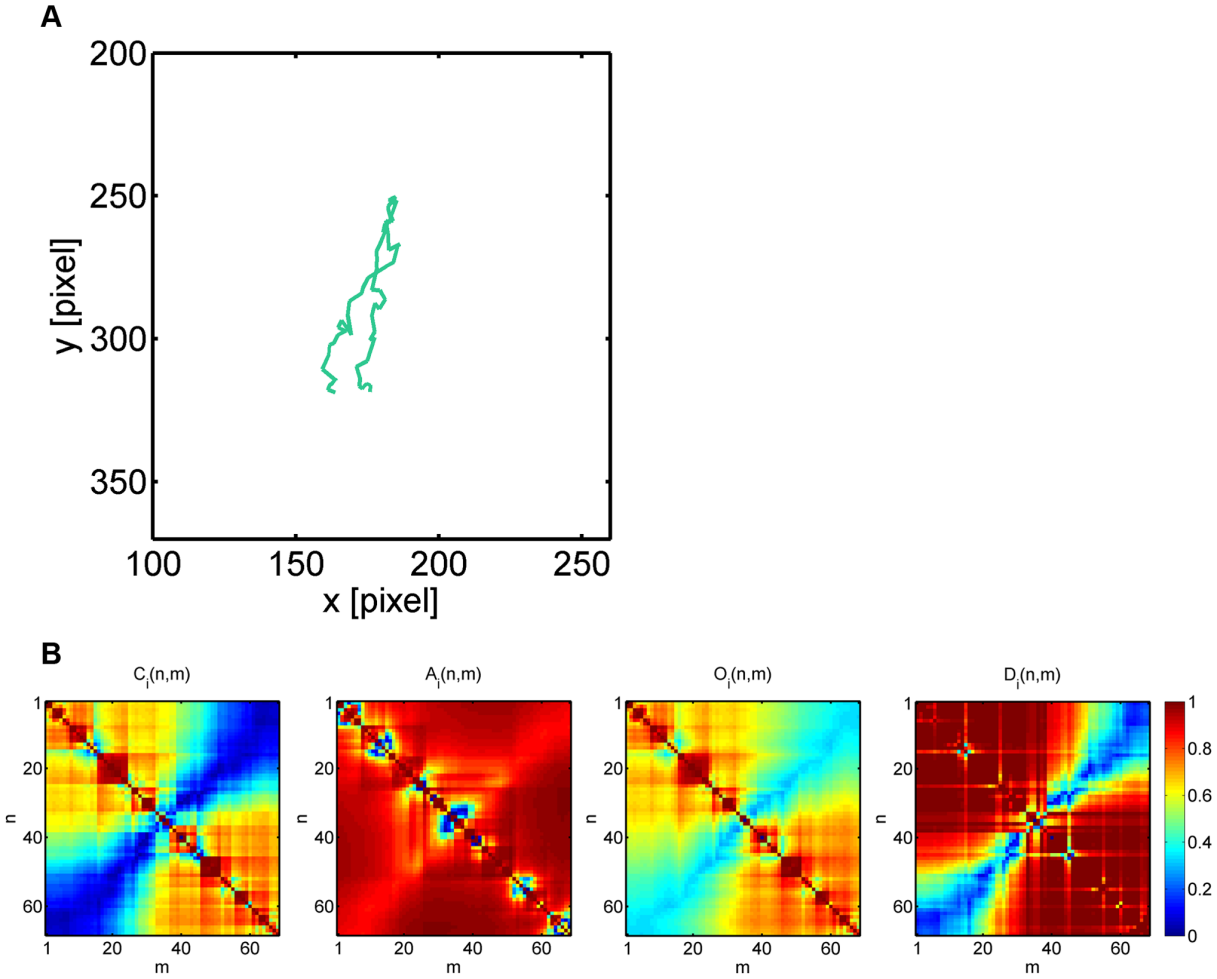
Neutrophil cell track with ID 245 switching sub-populations in going from 2D to 4D clustering. *(A)* Neutrophil with track ID 245 switched from sub-population of type 1 (fairly straight migration) to type 3 (random migration). *(B)* Heat maps of the staggered confinement ratio, staggered volume asphericity, staggered outreach ratio and staggered displacement ratio.

Going from 2D to 3D clustering, we generally observed that the numerical results got more stabilized and were already comparable to the values obtained from 4D clustering (see Figs. S7 and S8). To investigate whether higher-dimensional clustering induces reasonable adjustments of lower-dimensional clustering, we analyzed by way of example two cell tracks that switched sub-populations between 2D clustering based on the average staggered confinement ratio and the average staggered volume asphericity (CA clustering) and 4D clustering (CAOD clustering). These cell tracks are shown in Figs. 11 and 12 and are characterized by average staggered measures that are summarized in Table 5.

**Table 5:** Average staggered measures for two examples of neutrophil cell tracks that switch between sub-populations.

average staggered measures	track ID 1	track ID 245
confinement ratio	0.10	0.50
volume asphericity	0.62	0.87
outreach ratio	0.18	0.59
displacement ratio	0.47	0.78
2D 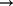 4D clustering	type 3 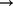 type 2	type 1 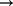 type 3

In going from 2D to 4D clustering, confinement ratio and volume asphericity are complemented by outreach ratio and displacement ratio that enforce switching between sub-populations of different types of migration behavior.

The neutrophil track with ID 1 was classified into the sub-population of randomly migrating cells (type 3) by CA clustering in 2D, even though it is obviously strongly confined (see Fig. 11): with more than four hours tracking time (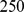 minutes) it was one of the longest cell tracks, while its maximal displacement remained well below 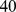 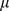
m. The neutrophil track with ID 245 also reached a maximal displacement in the order of 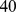 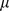
m, however, the tracking time was only 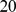 minutes in this case and it was classified into the sub-population of fairly straight cell tracks (type 1) by CA clustering (see Fig. 12), even though due to a large turning angle close to 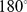 the end point of the track was only a few micrometers apart from its start point. The intuitive choice of sub-populations for these cells were automatically assigned by CAOD clustering in 4D: neutrophil track with ID 1 was classified as a strongly confined cell track (type 2) and neutrophil track with ID 245 switched to the sub-population of randomly migrating cells (type 3).

In both cases the reason for the misclassification in CA clustering was due to a high value in the average staggered volume asphericity (see Table 5), which is a direct consequence of the elongated shapes of these cell tracks (see Figs. 11 and 12). We recall that the volume asphericity completely neglects the time-ordering in the sequence of cell positions. Therefore, whatever a cell's exact type of migration is, elongated volumes of data points will receive a high asphericity score, which can compensate for a small confinement ratio and by that result in a misclassification. The impact of the volume apshericity is balanced by including other measures like the outreach ratio and the displacement ratio that together give a more accurate quantitative description of the cell track.

We conclude that the combination of staggered measures generally improved classification results towards higher-dimensional clustering. Quantitative discrepancies were mostly observed for different combinations of 2D clustering and the combination of the average staggered confinement ratio and the average staggered volume asphericity plays a key role in achieving reasonable clustering that can be further stabilized by higher-dimensional clustering.

## Discussion

The considerable progress in microscopy technologies during recent years enabled unprecedented exploration of biological systems, including the migration of cells that drives the dynamics of many biological processes. Image-based systems biology aims at quantitatively analysing and modeling these processes based on the information contained in microscopic image data. In this work we presented a novel approach to perform automated characterization and parameter-free classification of cell tracks at the single-cell level that avoids integrating out absolute cell positions in the biological sample and relative temporal offsets between cell tracks, which usually is the drawback of statistical analyses at the level of cell populations. At the heart of our approach is the discovery that by computing characteristic measures in a staggered fashion, *i.e.* separately for each possible segment of a cell track, we can characterize cell tracks based on the local information and can classify them into sub-populations with common properties from a combination of these measures.

In the present study, we focused on the following four measures that can be computed for cell tracks in any spatial dimension: (i) confinement ratio corresponding to the ratio of the displacement between two points over the length of the cell track between these points, (ii) volume asphericity characterizing the deviation of the cloud of uncorrelated cell positions in a track segment from a spherical volume, (iii) outreach ratio corresponding to the maximal displacement within a track segment over its length, and (iv) displacement ratio measuring the ratio of the displacement between the start and end point of a track segment over the maximal displacement contained in this segment. Viewing cell tracks as a way to probe the local environment in the biological sample, the local recognition of characteristic patterns can only be achieved by computing these measures in a staggered fashion, whereas this information gets obscured in the commonly performed computation of such measures as linear quantities. Other charming aspects of the staggered measures are that they are (i) representable by heat maps, (ii) straightforwardly interpretable, (iii) universally applicable and (iv) computationally cheap.

The superiority of staggered measures over linear measures can be explained as follows. Linear measures suffer from the burden that quantities at some time point are computed as the average over all previous time points. This implies that identical track segments occurring at different positions along the cell track give rise to different impact on the linear measures, since the track segment at the later time point enters the averaging relative to the initial time point with less weight. Therefore, with increasing number of time points linear measures lose sensitivity for temporal changes in the cell migration behavior. In contrast, computing staggered measures has the following advantage with regard to the characterization of cell migration: identical track segments within the cell track are resolved by their different starting points in the staggered measure and contribute to the average of the staggered measure with the same weight.

Most importantly, as was demonstrated both for synthetic cell track data generated *in silico* as well as for neutrophil track data from microscopy experiments, the average staggered measures convey information that can be combined and exploited to classify cell tracks into sub-populations with common properties. This is a direct consequence of the fact that these average values contain more local information on the cell track than in the case of averaging linear measures. Therefore, spanning a parameter space by the average staggered measures, cell tracks are represented as points that can be clustered by the parameter-free method of agglomerative hierarchical clustering. The resulting dendrogram uncovered the heterogeneity in the considered population of cells and three clusters in the continuum of migration behavior could be identified that corresponded to sub-populations with (i) fairly straight, (ii) strongly confined and (iii) purely random cell tracks. It should be noted that these short descriptions of sub-populations should be taken with some care and it is more appropriate referring to them as combinations of (i) high, (ii) low, and (iii) intermediate values in the average staggered measures, respectively.

We generally observed that the numerical results depend on the dimension of the parameter space and that clustering on only two average staggered measures requires a careful combination. In particular, combining the average staggered confinement ratio and the average staggered volume asphericity produced the best results for 2D clustering relative to 4D clustering involving all four staggered measures. Misclassifications occurred in a few percent of cases when cell tracks happened to cover fairly elongated volumes while the local migration behavior was quite random. These rare events induced a dilemma between the average staggered confinement ratio and the average staggered volume asphericity that could, however, be resolved by increasing the dimension of the parameter space.

Finally, it is possible to exploit the information of a staggered measure contained in its heat map in order to study dynamic changes in the migration behavior of single cells. Mapping clusters of cell tracks with similar migration behavior back into a system's spatial environment allows drawing conclusions on the structure and morphology of biological samples. The general formulation of our approach promotes its broad application to tracks of arbitrary objects and its straightforward extension with regard to other staggered measures that may further improve the results on the automated characterization and parameter-free classification of tracks.

## Supporting Information

Figure S1 **Cell track population of type 1.** *(A)* Examples of cell tracks. *(B)* Instantaneous speed distribution. *(C)* Turning angle distribution.
(TIF)

Figure S2 **Cell track population of type 2.** *(A)* Examples of cell tracks. *(B)* Instantaneous speed distribution. *(C)* Turning angle distribution.
(TIF)

Figure S3 **Cell track population of type 3.** *(A)* Examples of cell tracks. *(B)* Instantaneous speed distribution. *(C)* Turning angle distribution.
(TIF)

Figure S4 **Volume prolateness for populations and single cell tracks.** The dependence of the volume prolateness on time is comparable to that of the volume asphericity. *(A)* Population of all cell tracks (grey), type 1 population (red), type 2 population (green) and type 3 population (blue) (see Fig. 3B). Error bars correspond to the standard deviation and are only shown at selected time points to enhance clarity. *(B)* Selected cell tracks of each population type (see Figs. 4 and 5B).
(TIF)

Figure S5 **Staggered volume prolateness for single cell tracks.** The dependence of the staggered volume prolateness on time is comparable to that of the staggered volume asphericity (see Fig. 6).
(TIF)

Figure S6 **Clustering of synthetic cell track data in the parameter space of average linear measures.** *(A)* Synthetic cell track data in the the parameter space of the average linear confinement ratio and the average linear volume asphericity. Red, green and blue color refer to the three sub-populations obtained from hierarchical clustering. *(B)* Representation of synthetic cell track data in the parameter space of average staggered measures as obtained from hierarchical clustering in the parameter space of average linear measures (see Fig. 7).
(TIF)

Figure S7 **Average staggered measures of neutrophil tracks per sub-population for 2D, 3D and 4D clustering.** The method of clustering is abbreviated by the initial C for confinement ratio, A for volume asphericity, O for outreach ratio and D for displacement ratio. Each averaged staggered measure is plotted for sub-population of type 1 (red), type 2 (green) and type 3 (blue). *(A)* Average staggered confinement ratio. *(B)* Average staggered volume asphericity. *(C)* Average staggered outreach ratio. *(D)* Average staggered displacement ratio.
(TIF)

Figure S8 **Number of neutrophil tracks per sub-population for 2D, 3D and 4D clustering.** The method of clustering is abbreviated by the initial C for confinement ratio, A for volume asphericity, O for outreach ratio and D for displacement ratio. Number of neutrophil cell tracks for sub-population of type 1 (red), type 2 (green) and type 3 (blue).
(TIF)

Figure S9
**Cell population analyses obtained by 4D clustering for 119 fairly straight neutrophil cell tracks (type 1).** *(A)* Instantaneous speed distribution with average speed 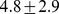 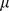
m/min. *(B)* Turning angle distribution with average angle 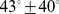. *(C)* Displacement curve showing quadratic dependence on the square-root of time. Error bars correspond to the standard deviation and are only shown at selected time points to enhance clarity.(TIF)

Figure S10
**Cell population analyses obtained by 4D clustering for 22 strongly confined neutrophil cell tracks (type 2).** *(A)* Instantaneous speed distribution with average speed 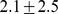 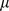
m/min. *(B)* Turning angle distribution with average angle 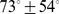. *(C)* Displacement curve showing linear dependence on the square-root of time. Error bars correspond to the standard deviation and are only shown at selected time points to enhance clarity.(TIF)

Figure S11
**Cell population analyses obtained by 4D clustering for 150 purely random neutrophil cell tracks (type 3).** *(A)* Instantaneous speed distribution with average speed 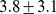 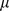
m/min. *(B)* Turning angle distribution with average angle 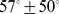. *(C)* Displacement curve showing linear dependence on the square-root of time. Error bars correspond to the standard deviation and are only shown at selected time points to enhance clarity.(TIF)

Figure S12 **Cell track data in the parameter space of average linear measures.** Cell track data in the the parameter space of the average linear confinement ratio and the average linear volume asphericity. Red, green and blue color refer to the cell migration types 1, 2, and 3, respectively, that were previously obtained from the clustering in the parameter space of average staggered measures (see Fig. 9).
(TIF)

Movie S1 **Time-lapse microscopy experiment of** ***in vitro*** **neutrophil migration.**
(AVI)

Table S1 **Quantitative evaluation of clustering results for synthetic cell track data in the parameter space of average linear measures.**
(PDF)

## References

[1] Antony PMA, Trefois C, Stojanovic A, Baumuratov AS, Kozak K (2013) Light microscopy applications in systems biology: Opportunities and challenges. Cell Communication and Signaling 11: 24. 23578051 10.1186/1478-811X-11-24 PMC3627909

[2] Meijering E, Dzyubachyk O, Smal I (2012) Methods for cell and particle tracking. In: Methods in Enzymology, Academic Press, volume 504, chapter 9: 183–200 doi:10.1016/B978-0-12-391857-4.00009-4 10.1016/B978-0-12-391857-4.00009-4 22264535

[3] Zimmer C (2012) From microbes to numbers: Extracting meaningful quantities from images. Cellular Microbiology 14: 1828–1835. 22985180 10.1111/cmi.12032

[4] Allen C, Okada T, Tang H, Cyster J (2007) Imaging of germinal center selection events during affinity maturation. Science Signalling 315: 528–531. 10.1126/science.1136736 17185562

[5] Schwickert T, Lindquist R, Shakhar G, Livshits G, Skokos D, et al (2007) In vivo imaging of germinal centres reveals a dynamic open structure. Nature 446: 83–87. 17268470 10.1038/nature05573

[6] Hauser A, Junt T, Mempel T, Sneddon M, Kleinstein S, et al (2007) Definition of germinal-center B cell migration in vivo reveals predominant intrazonal circulation patterns. Immunity 26: 655–667. 17509908 10.1016/j.immuni.2007.04.008

[7] Figge M, Garin A, Gunzer M, Kosco-Vilbois M, Toellner K, et al (2008) Deriving a germinal center lymphocyte migration model from two-photon data. The Journal of experimental medicine 205: 3019–3029. 19047437 10.1084/jem.20081160 PMC2605235

[8] Beltman J, Allen C, Cyster J, de Boer R (2011) B cells within germinal centers migrate preferentially from dark to light zone. Proceedings of the National Academy of Sciences 108: 8755–8760. 10.1073/pnas.1101554108 PMC3102384 21555569

[9] Meyer-Hermann M, Figge M, Toellner K (2009) Germinal centres seen through the mathematical eye: B-cell models on the catwalk. Trends in immunology 30: 157–164. 19282244 10.1016/j.it.2009.01.005

[10] Weber M, Hauschild R, Schwarz J, Moussion C, de Vries I, et al (2013) Interstitial dendritic cell guidance by haptotactic chemokine gradients. Science 339: 328–332. 23329049 10.1126/science.1228456

[11] Coelho FM, Natale D, Soriano SF, Hons M, Swoger J, et al (2013) Naïve B cell trafficking is shaped by local chemokine availability and lfa-1-independent stromal interactions. Blood 121: 4101–4109. 23558016 10.1182/blood-2012-10-465336

[12] Sarris M, Masson JB, Maurin D, Van der Aa LM, Boudinot P, et al (2012) Inammatory chemokines direct and restrict leukocyte migration within live tissues as glycan-bound gradients. Current Biology 22: 2375–2382. 23219724 10.1016/j.cub.2012.11.018

[13] Sumen C, Mempel TR, Mazo IB, von Andrian UH (2004) Intravital microscopy: visualizing immunity in context. Immunity 21: 315–329. 15357943 10.1016/j.immuni.2004.08.006

[14] Huet S, Karatekin E, Tran V, Fanget I, Cribier S, et al (2006) Analysis of transient behavior in complex trajectories: application to secretory vesicle dynamics. Biophysical journal 91: 3542–3559. 16891360 10.1529/biophysj.105.080622 PMC1614485

[15] Blavatska V, von Ferber C, Holovatch Y (2011) Shapes of macromolecules in good solvents: field theoretical renormalization group approach. Condensed Matter Physics 14: 33701–1–33701–20.

[16] Beltman JB, Marée AF, de Boer RJ (2009) Analysing immune cell migration. Nature Reviews Immunology 9: 789–798. 10.1038/nri2638 19834485

[17] Vedel S, Tay S, Johnston DM, Bruus H, Quake SR (2013) Migration of cells in a social context. Proceedings of the National Academy of Sciences 110: 129–134. 10.1073/pnas.1204291110 PMC3538227 23251032

[18] Press W (2007) Numerical Recipes 3rd Edition: The Art of Scientific Computing. Cambridge University Press.

[19] Hasenberg M, Köhler A, Bonifatius S, Borucki K, Riek-Burchardt M, et al (2011) Rapid immunomagnetic negative enrichment of neutrophil granulocytes from murine bone marrow for functional studies *in vitro* and *in vivo* . PLoS ONE 6: e17314. 21383835 10.1371/journal.pone.0017314 PMC3044161

[20] Abramoff M, Magalhaes P, Ram S (2004) Image processing with ImageJ. Biophotonics International 11: 36–42.

[21] Figge M, Meyer-Hermann M (2011) Modelling intravital two-photon data of lymphocyte migration and interaction. Mathematical Models and Immune Cell Biology 978-1-4419-7724-3: 121–139.

