# Correction: Automated Characterization and Parameter-Free Classification of Cell Tracks Based on Local Migration Behavior

**DOI:** 10.1371/journal.pone.0115158

**Published:** 2014-12-04

**Authors:** The PLOS ONE Staff

In the Methods section, Equations 10 and 11 are missing due to a typesetting error. The publisher apologizes for this error. The equations can be found in the correct context below:

“where we assume without loss of generality that 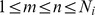. The track length between time points 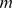 and 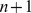 can be represented as
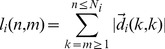
(10)in terms of the displacement vector 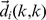 that refers to subsequent time points 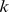 and 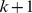. The staggered confinement ratio is then defined as the ratio of these two quantities,
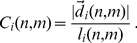
(11)


Viewing 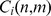 as entries of the 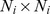 matrix 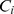, we note that this matrix is symmetric because both the displacement vector 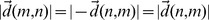 and the track-segment length 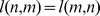 are invariant under the time reversal operation 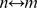 such that 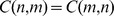. Furthermore, the diagonal elements of 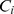 take values 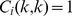 because 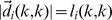 for all 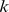. In general, 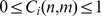, since”

## References

[1] Mokhtari Z, Mech F, Zitzmann C, Hasenberg M, Gunzer M, et al (2013) Automated Characterization and Parameter-Free Classification of Cell Tracks Based on Local Migration Behavior. PLoS ONE 8(12): e80808 doi:10.1371/journal.pone.0080808 24324630 10.1371/journal.pone.0080808 PMC3855794

