# Supplementary figures and images for: Automated Characterization and Parameter-Free Classification of Cell Tracks Based on Local Migration Behavior
Source: PLoS One. 2013 Dec 6;8(12):e80808. doi: 10.1371/journal.pone.0080808 (PMC3855794; doi:10.1371/journal.pone.0080808)

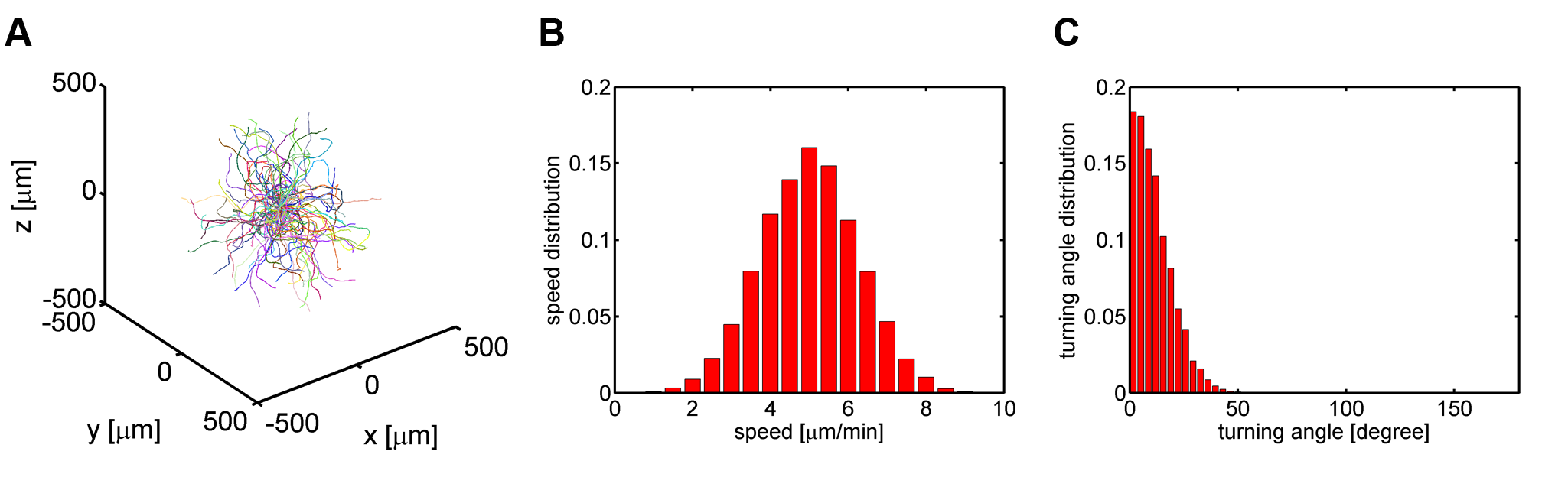

Supplement: Figure S1 — Cell track population of type 1. (A) Examples of cell tracks. (B) Instantaneous speed distribution. (C) Turning angle distribution. (TIF) [file pone.0080808.s001.tif]

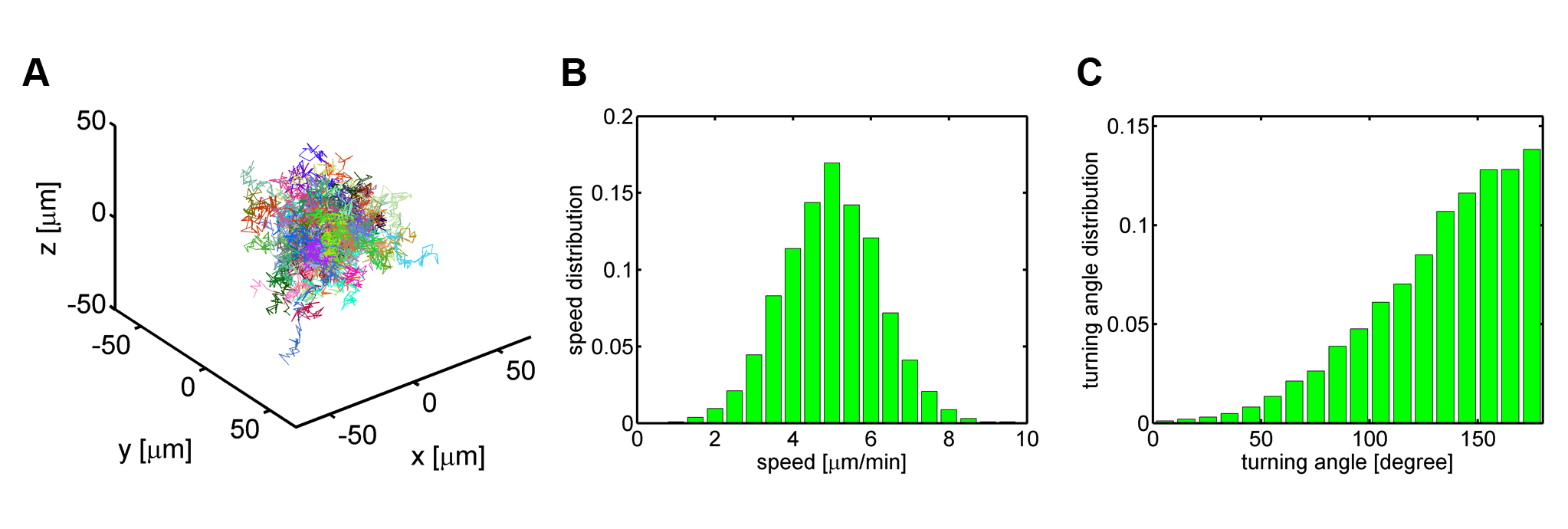

Supplement: Figure S2 — Cell track population of type 2. (A) Examples of cell tracks. (B) Instantaneous speed distribution. (C) Turning angle distribution. (TIF) [file pone.0080808.s002.tif]

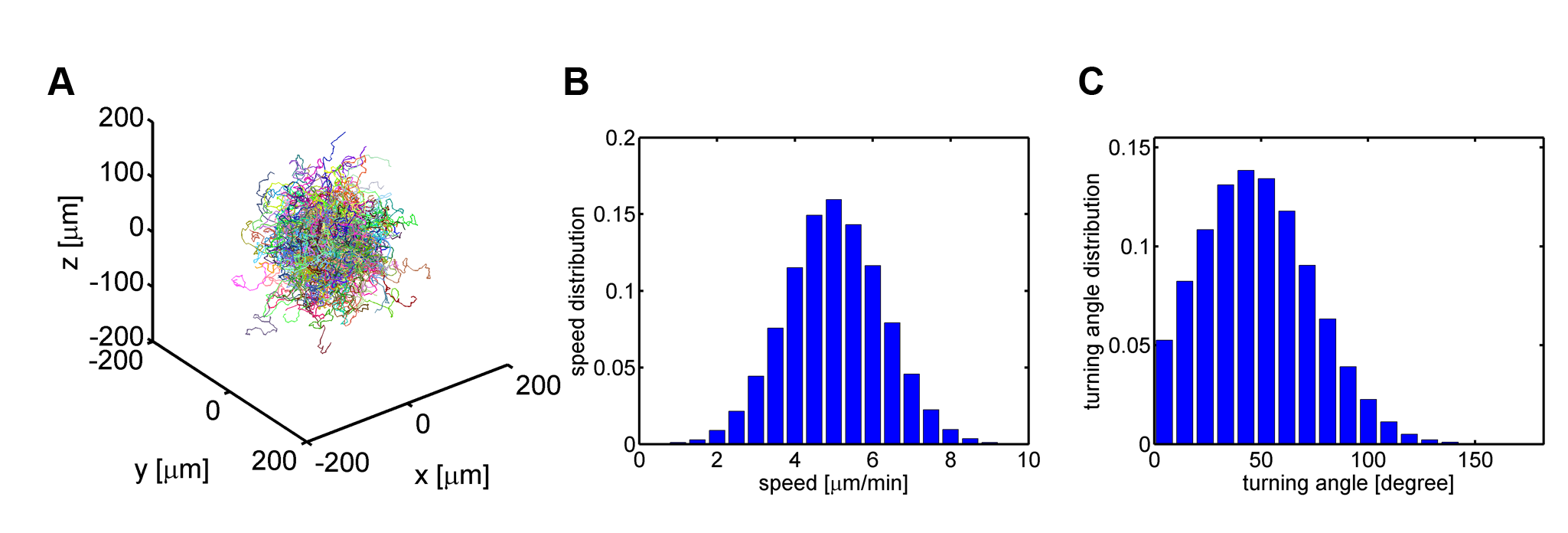

Supplement: Figure S3 — Cell track population of type 3. (A) Examples of cell tracks. (B) Instantaneous speed distribution. (C) Turning angle distribution. (TIF) [file pone.0080808.s003.tif]

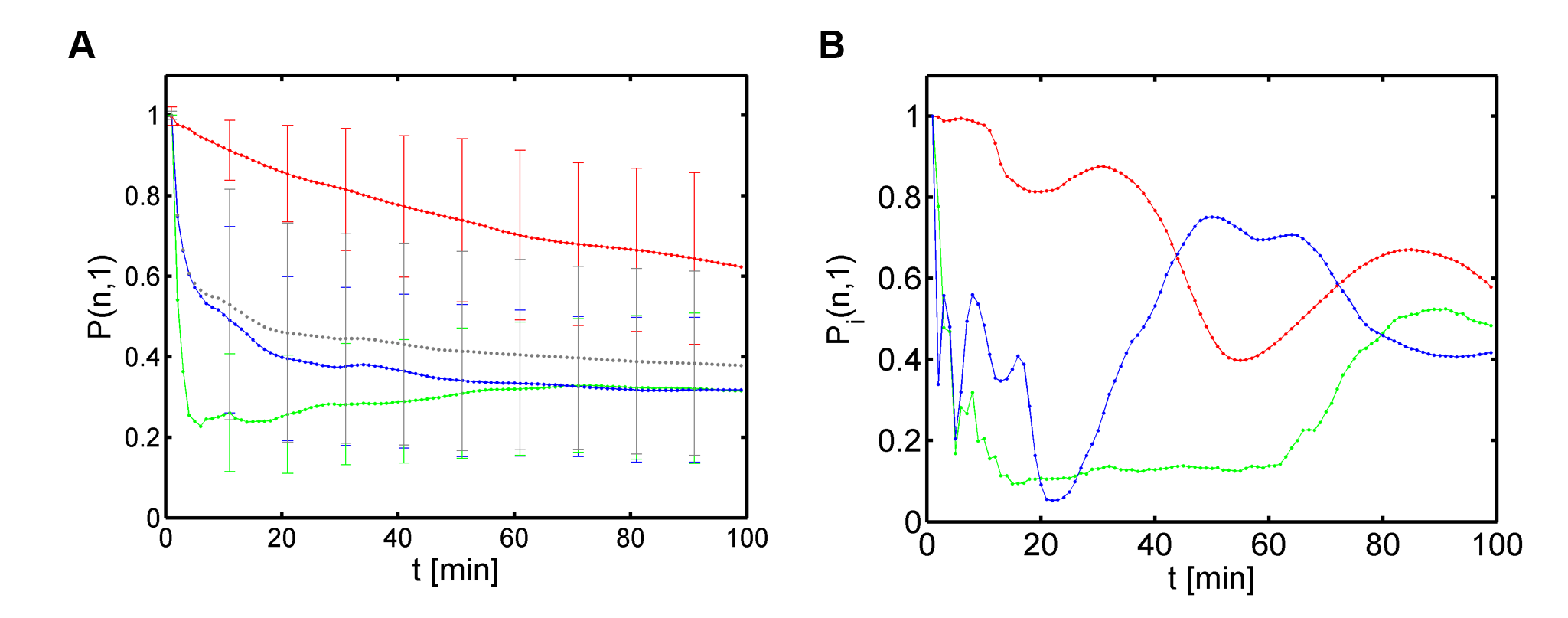

Supplement: Figure S4 — Volume prolateness for populations and single cell tracks. The dependence of the volume prolateness on time is comparable to that of the volume asphericity. (A) Population of all cell tracks (grey), type 1 population (red), type 2 population (green) and type 3 population (blue) (see Fig. 3B). Error bars correspond to the standard deviation and are only shown at selected time points to enhance clarity. (B) Selected cell tracks of each population type (see Figs. 4 and 5B). (TIF) [file pone.0080808.s004.tif]

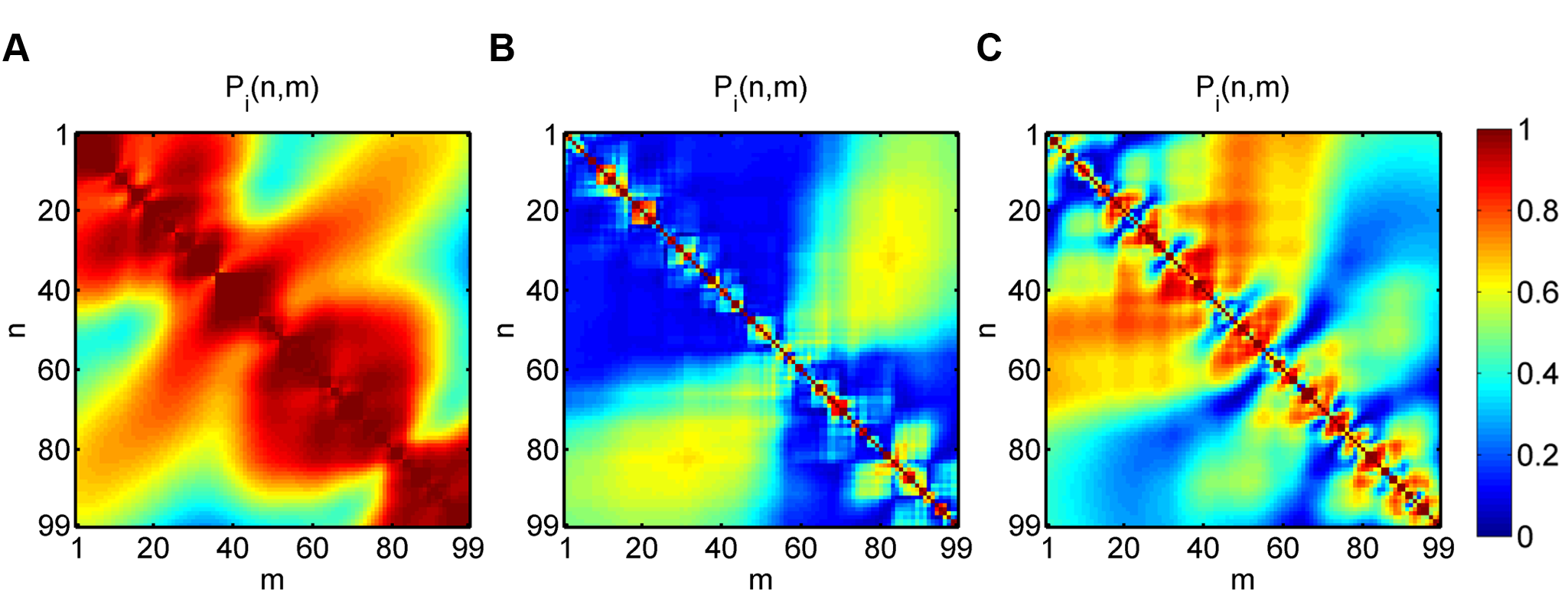

Supplement: Figure S5 — Staggered volume prolateness for single cell tracks. The dependence of the staggered volume prolateness on time is comparable to that of the staggered volume asphericity (see Fig. 6). (TIF) [file pone.0080808.s005.tif]

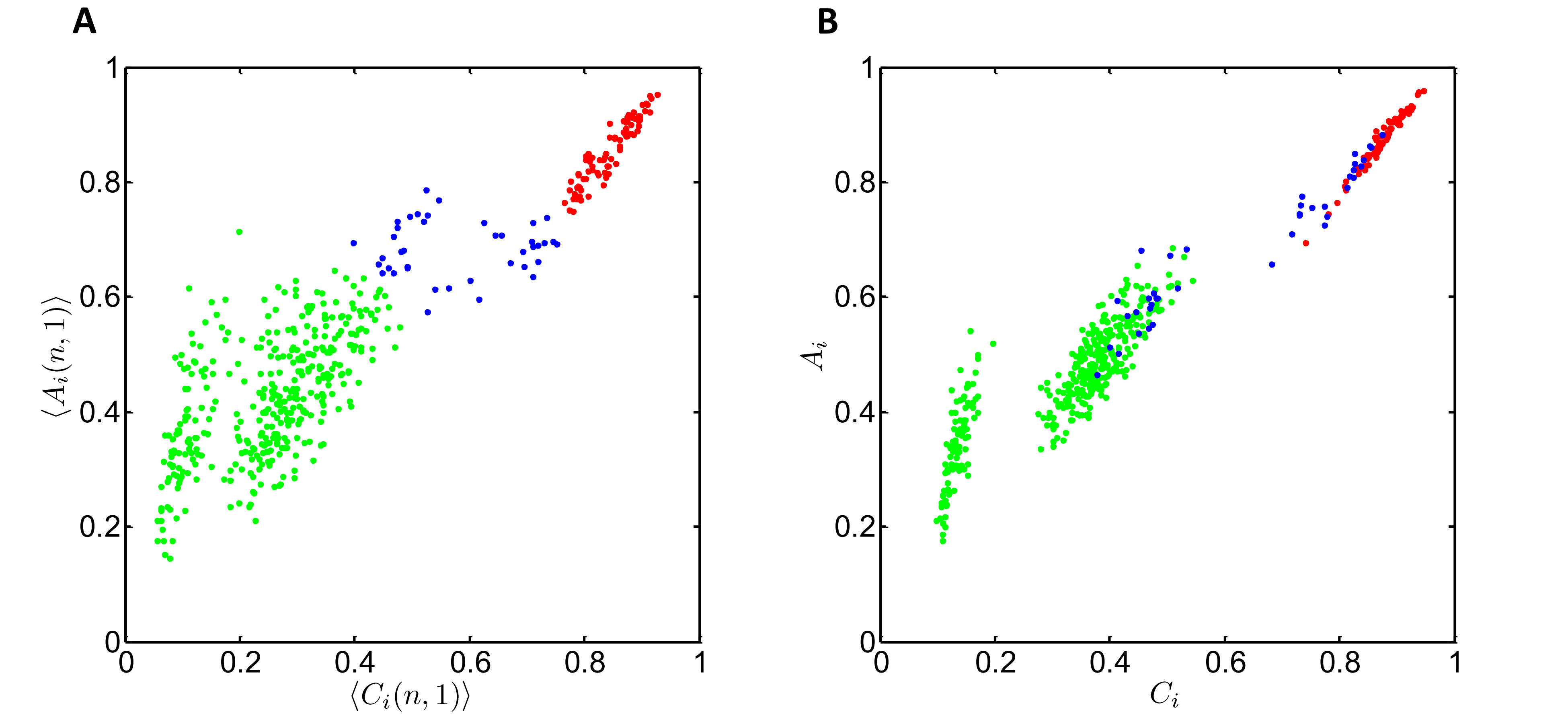

Supplement: Figure S6 — Clustering of synthetic cell track data in the parameter space of average linear measures. (A) Synthetic cell track data in the the parameter space of the average linear confinement ratio and the average linear volume asphericity. Red, green and blue color refer to the three sub-populations obtained from hierarchical clustering. (B) Representation of synthetic cell track data in the parameter space of average staggered measures as obtained from hierarchical clustering in the parameter space of average linear measures (see Fig. 7). (TIF) [file pone.0080808.s006.tif]

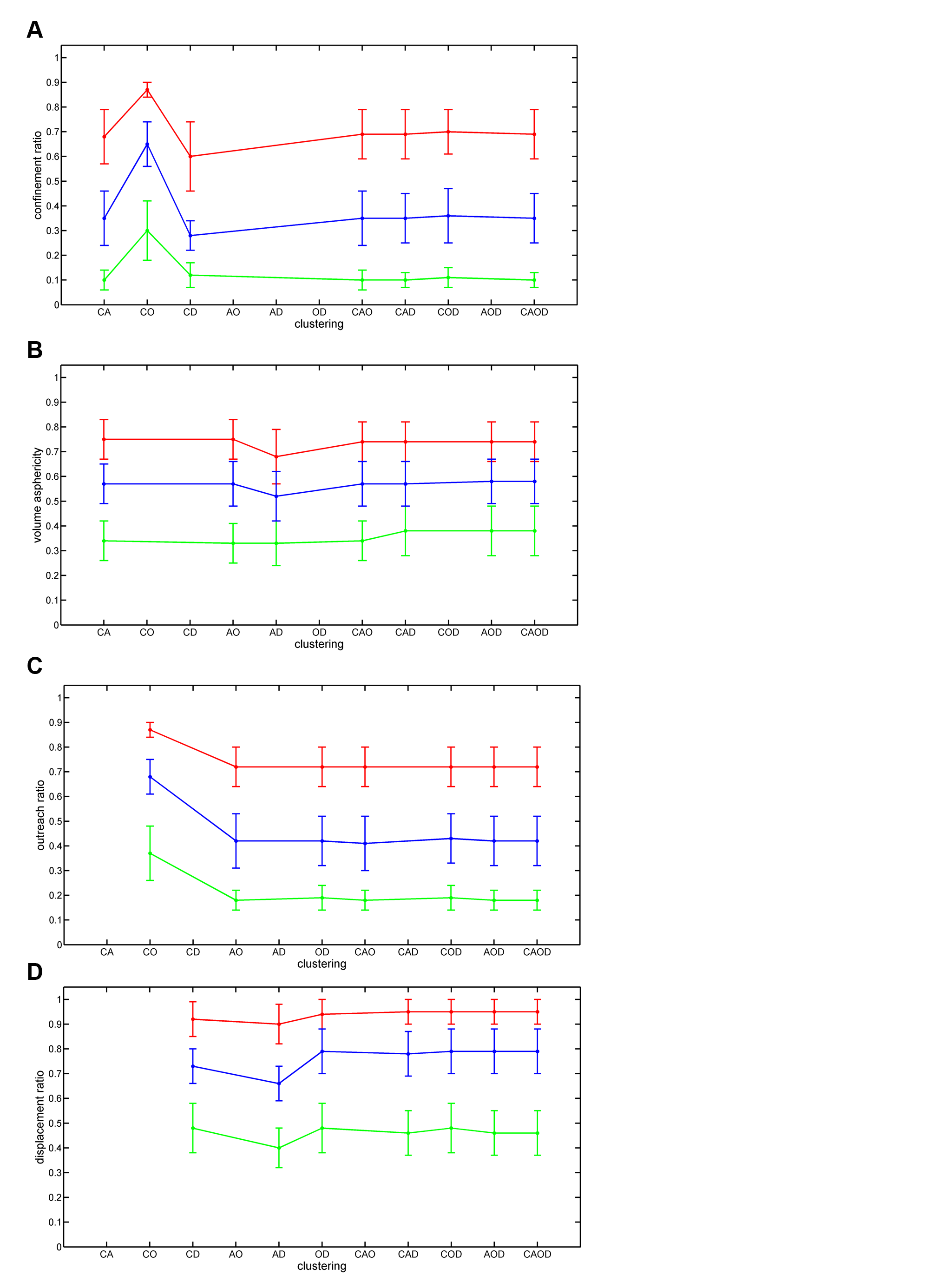

Supplement: Figure S7 — Average staggered measures of neutrophil tracks per sub-population for 2D, 3D and 4D clustering. The method of clustering is abbreviated by the initial C for confinement ratio, A for volume asphericity, O for outreach ratio and D for displacement ratio. Each averaged staggered measure is plotted for sub-population of type 1 (red), type 2 (green) and type 3 (blue). (A) Average staggered confinement ratio. (B) Average staggered volume asphericity. (C) Average staggered outreach ratio. (D) Average staggered displacement ratio. (TIF) [file pone.0080808.s007.tif]

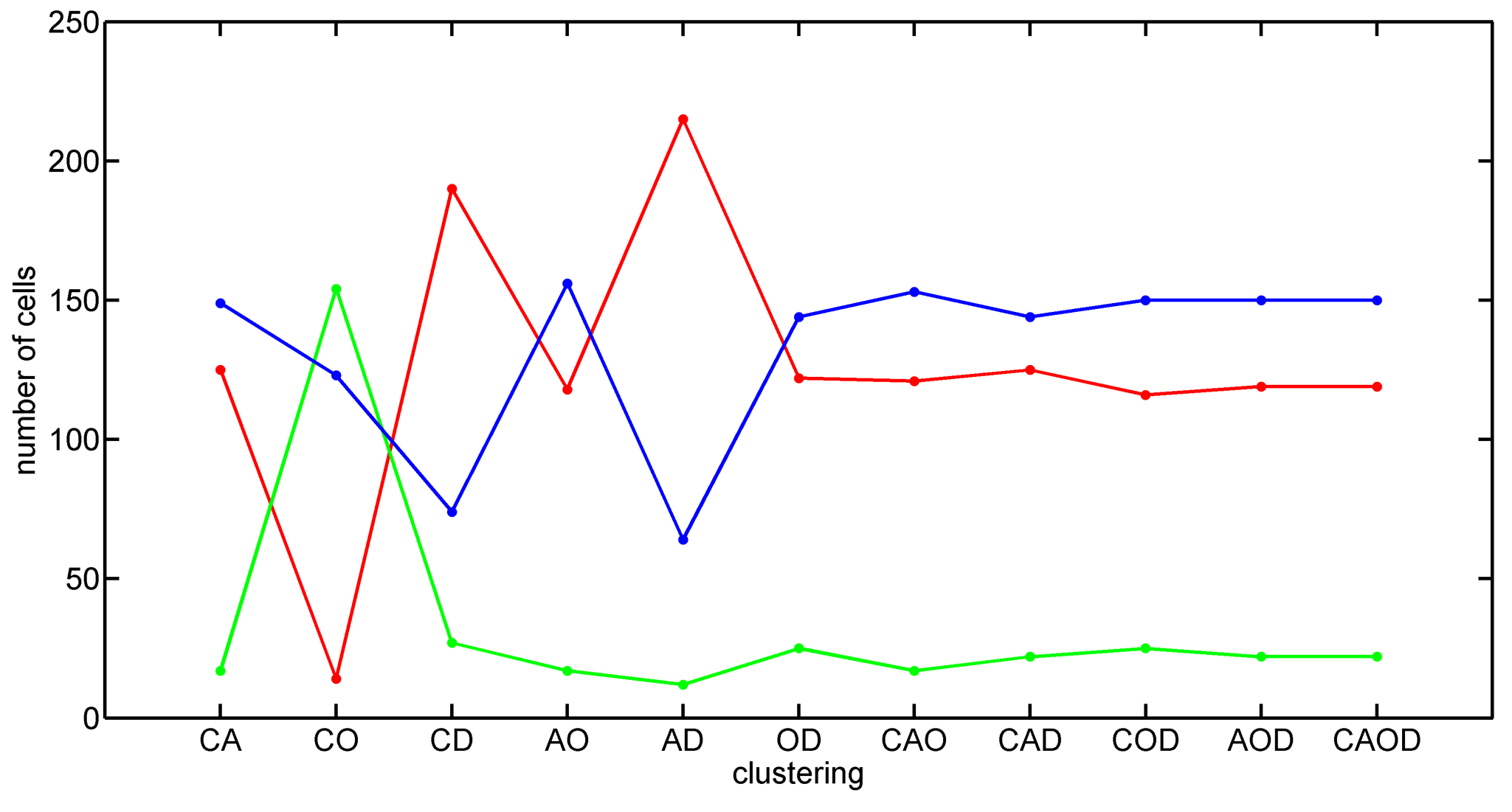

Supplement: Figure S8 — Number of neutrophil tracks per sub-population for 2D, 3D and 4D clustering. The method of clustering is abbreviated by the initial C for confinement ratio, A for volume asphericity, O for outreach ratio and D for displacement ratio. Number of neutrophil cell tracks for sub-population of type 1 (red), type 2 (green) and type 3 (blue). (TIF) [file pone.0080808.s008.tif]

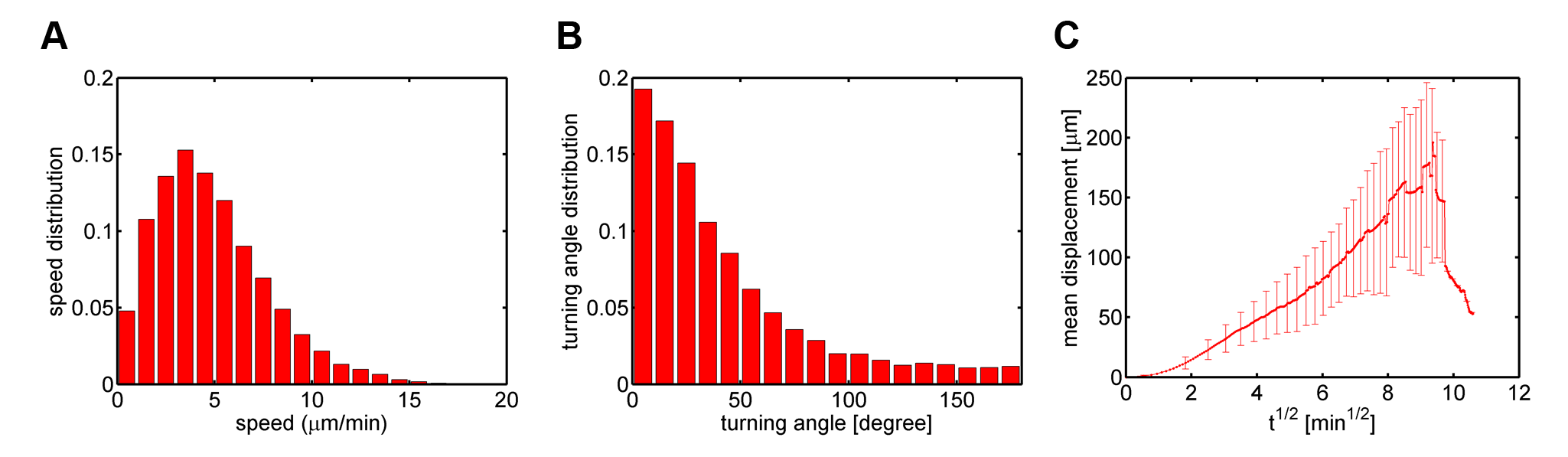

Supplement: Figure S9 — Cell population analyses obtained by 4D clustering for 119 fairly straight neutrophil cell tracks (type 1). (A) Instantaneous speed distribution with average speed m/min. (B) Turning angle distribution with average angle . (C) Displacement curve showing quadratic dependence on the square-root of time. Error bars correspond to the standard deviation and are only shown at selected time points to enhance clarity. (TIF) [file pone.0080808.s009.tif]

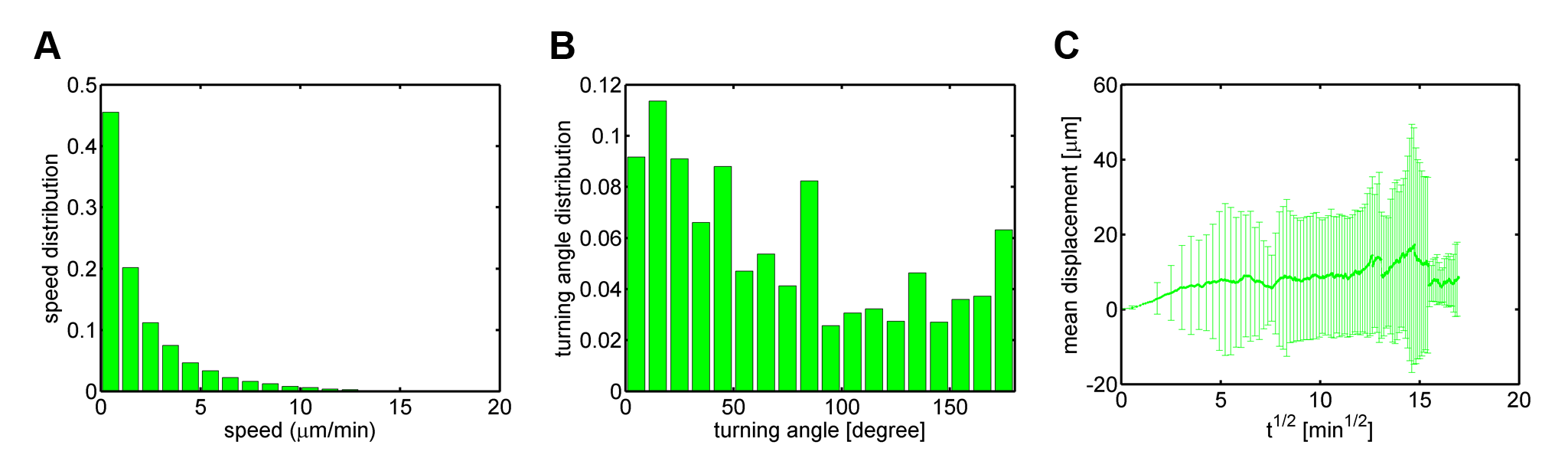

Supplement: Figure S10 — Cell population analyses obtained by 4D clustering for 22 strongly confined neutrophil cell tracks (type 2). (A) Instantaneous speed distribution with average speed m/min. (B) Turning angle distribution with average angle . (C) Displacement curve showing linear dependence on the square-root of time. Error bars correspond to the standard deviation and are only shown at selected time points to enhance clarity. (TIF) [file pone.0080808.s010.tif]

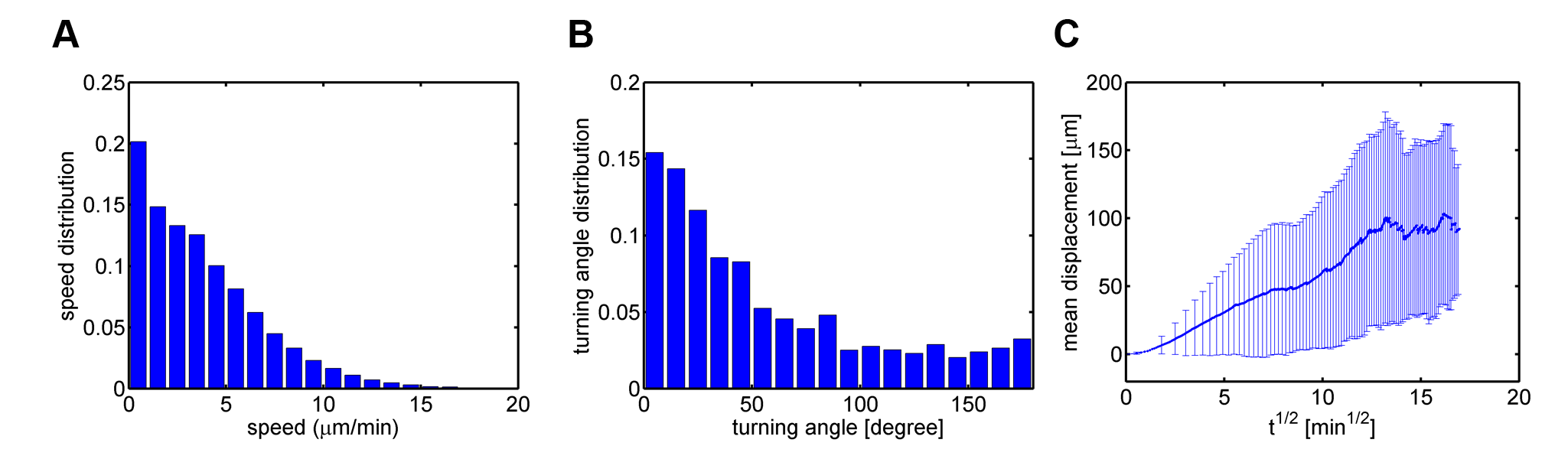

Supplement: Figure S11 — Cell population analyses obtained by 4D clustering for 150 purely random neutrophil cell tracks (type 3). (A) Instantaneous speed distribution with average speed m/min. (B) Turning angle distribution with average angle . (C) Displacement curve showing linear dependence on the square-root of time. Error bars correspond to the standard deviation and are only shown at selected time points to enhance clarity. (TIF) [file pone.0080808.s011.tif]

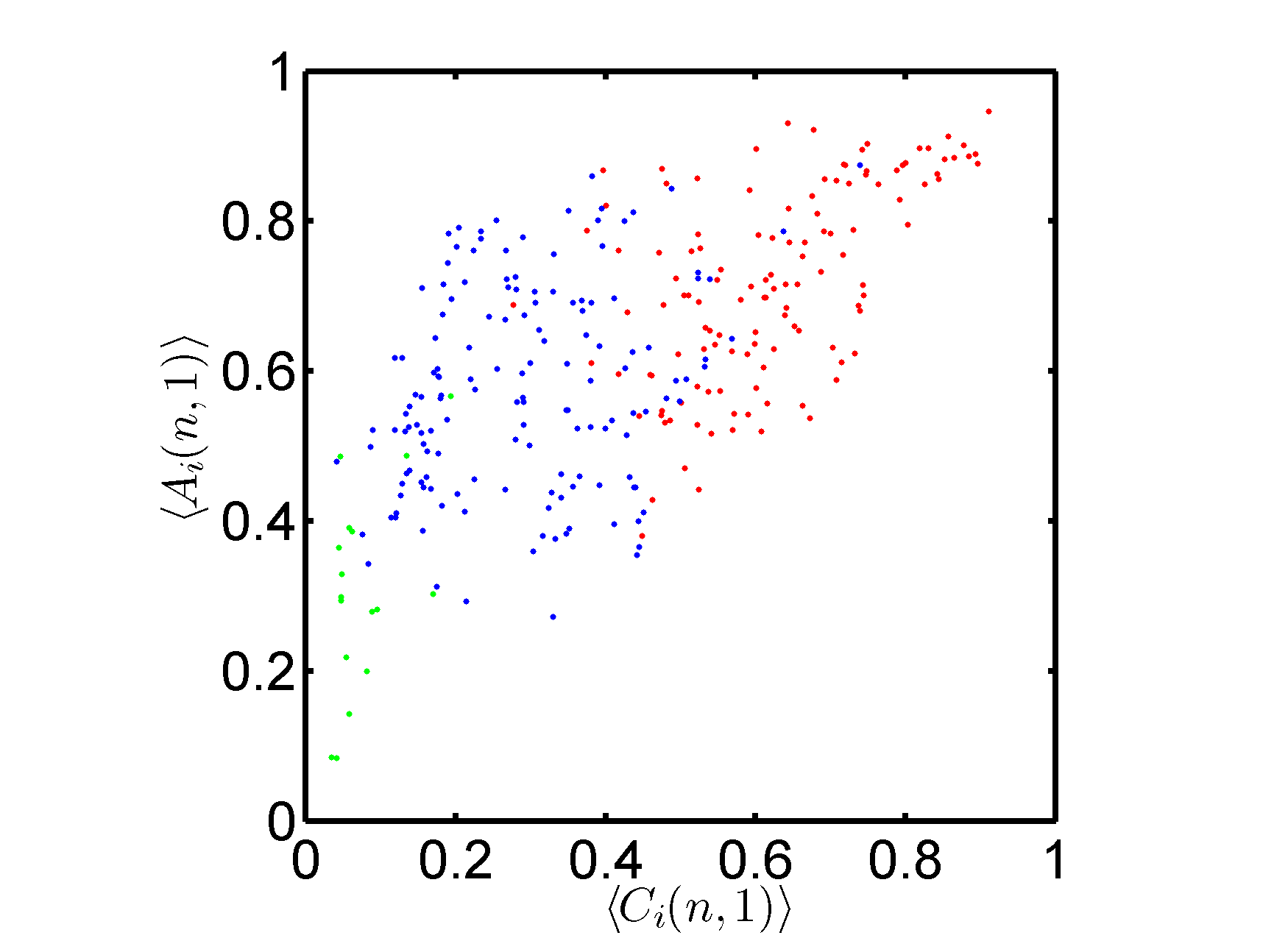

Supplement: Figure S12 — Cell track data in the parameter space of average linear measures. Cell track data in the the parameter space of the average linear confinement ratio and the average linear volume asphericity. Red, green and blue color refer to the cell migration types 1, 2, and 3, respectively, that were previously obtained from the clustering in the parameter space of average staggered measures (see Fig. 9). (TIF) [file pone.0080808.s012.tif]
