# Supplementary material for: Automated Characterization and Parameter-Free Classification of Cell Tracks Based on Local Migration Behavior
Source: PLoS One. 2013 Dec 6;8(12):e80808. doi: 10.1371/journal.pone.0080808 (PMC3855794; doi:10.1371/journal.pone.0080808)
Supplement: Table S1 — Quantitative evaluation of clustering results for synthetic cell track data in the parameter space of average linear measures. (PDF) [file pone.0080808.s014.pdf]

Table S1. Quantitative evaluation of clustering results for synthetic cell track data in the parameter space of average linear measures

measures	type 1: fairly straight	type 2: strongly confined	type 3: purely random
TP	79	100	19
FP	0	281	21
TN	400	119	179
FN	21	0	281
A	0.958	0.438	0.396
P	1	0.262	0.475
S	1	0.298	0.895
R	0.79	1	0.063

The clustering for synthetic cell track data in the parameter space of average linear measures as presented in Fig. S6 was evaluated by computing the true positives (TP), false positives (FP), true negatives (TN) and false negatives (FN) of the classification. The classification measures accuracy A=(TP+TN)/(TP+FP+TN+FN), precision P=TP/(TP+FP), specificity S=TN/(TN+FP) and recall R=TP/(TP+FN) are presented for each of the three types of migration. The values should be compared with the relevant quantities obtained from clustering in the parameter space of average staggered measures that yield A=P=S=R=1 since FP=FN=0 for each of the three types of migration.
